# Multiomic Signatures of Traffic-Related Air Pollution in London Reveal Potential Short-Term Perturbations in Gut Microbiome-Related Pathways

**DOI:** 10.1021/acs.est.3c09148

**Published:** 2024-05-10

**Authors:** Sibo Lucas Cheng, Michael Hedges, Pekka Keski-Rahkonen, Anastasia Chrysovalantou Chatziioannou, Augustin Scalbert, Kian Fan Chung, Rudy Sinharay, David C. Green, Theo M. C. M. de Kok, Jelle Vlaanderen, Soterios A. Kyrtopoulos, Frank Kelly, Lützen Portengen, Paolo Vineis, Roel C. H. Vermeulen, Marc Chadeau-Hyam, Sonia Dagnino

**Affiliations:** † NIHR HPRU in Environmental Exposures and Health, Imperial College London, London W12 0BZ, U.K.; ‡ MRC Centre for Environment and Health, Department of Epidemiology and Biostatistics, School of Public Health, Imperial College London, London W12 7TA, U.K.; § MRC Centre for Environment and Health, Environmental Research Group, Imperial College London, London W12 0BZ, U.K.; ∥ International Agency for Research on Cancer (IARC), Lyon 69366 Cedex, France; ⊥ National Heart & Lung Institute, Imperial College London, London SW7 2AZ, U.K.; # Royal Brompton & Harefield NHS Trust, London SW3 6NP, U.K.; ∇ Imperial College Healthcare NHS Trust, London W2 1NY, U.K.; ○ Department of Toxicogenomics, GROW School for Oncology and Reproduction, Maastricht University, Maastricht 6229 ER, The Netherlands; ◆ Division of Environmental Epidemiology, Institute for Risk Assessment Sciences, Utrecht University, Utrecht 3584 CS, The Netherlands; ¶ National Hellenic Research Foundation, Athens 11635, Greece; †† Julius Centre for Health Sciences and Primary Care, University Medical Centre, Utrecht University, Utrecht 3584 CG, The Netherlands; ‡‡ Transporters in Imaging and Radiotherapy in Oncology (TIRO), School of Medicine, Direction de la Recherche Fondamentale (DRF), Institut des Sciences du Vivant Fréderic Joliot, Commissariat à l’Energie Atomique et aux Énergies Alternatives (CEA), Université Côte d’Azur (UniCA), Nice 06107, France

**Keywords:** air pollution, exposome, multiomics, metabolomics, tryptophan

## Abstract

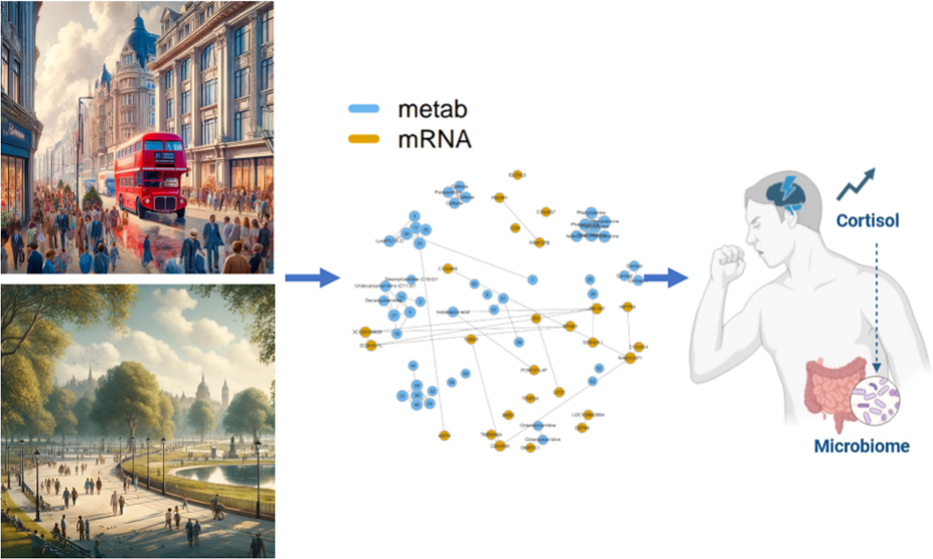

This randomized crossover study investigated the metabolic and mRNA alterations associated with exposure to high and low traffic-related air pollution (TRAP) in 50 participants who were either healthy or were diagnosed with chronic pulmonary obstructive disease (COPD) or ischemic heart disease (IHD). For the first time, this study combined transcriptomics and serum metabolomics measured in the same participants over multiple time points (2 h before, and 2 and 24 h after exposure) and over two contrasted exposure regimes to identify potential multiomic modifications linked to TRAP exposure. With a multivariate normal model, we identified 78 metabolic features and 53 mRNA features associated with at least one TRAP exposure. Nitrogen dioxide (NO_2_) emerged as the dominant pollutant, with 67 unique associated metabolomic features. Pathway analysis and annotation of metabolic features consistently indicated perturbations in the tryptophan metabolism associated with NO_2_ exposure, particularly in the gut-microbiome-associated indole pathway. Conditional multiomics networks revealed complex and intricate mechanisms associated with TRAP exposure, with some effects persisting 24 h after exposure. Our findings indicate that exposure to TRAP can alter important physiological mechanisms even after a short-term exposure of a 2 h walk. We describe for the first time a potential link between NO_2_ exposure and perturbation of the microbiome-related pathways.

## Abstract 2

This work represents a pioneering study combining transcriptomics and metabolomics in the same participants over multiple time points and two contrasted exposure regimes to investigate the effects of air pollution exposure.

## Introduction

Traffic-related air pollution (TRAP) emitted from motor vehicle tailpipe emissions and brake and tire wear is a major source of urban air pollution.^1,2^ TRAP comprises a complex mixture of gases and particles including nitrogen oxides (NO_2_ and NO_*x*_), particulate matter PM_10_ and PM_2.5_, particles with diameters less than or equal to 10 or 2.5 μm, respectively, ultrafine particles (particles of diameters less than or equal to 100 nm), and black carbon (BC). TRAP contributes to 25–40% of the ambient levels of major air pollutants.^2,3^

Epidemiological evidence has increasingly linked human exposure to urban particulate matter (PM) with serious adverse health effects, including increased mortality and morbidity.^4−6^ PMs, especially fine PM (PM_2.5_), may exert adverse health effects even at low exposures.^7^ Moreover, elevated levels of PM_2.5_ are strongly correlated with increased chronic obstructive pulmonary disease (COPD) and cardiovascular disease prevalence.^8−10^ Although the underlying mechanisms are poorly understood, it appears that the triggering of oxidative stress and inflammatory markers are fundamental to increasing risk.^11−14^ Human health studies could reveal mechanistic processes and distinct end points associated with short- and long-term exposure to TRAP-related pollutants.^15^ Such studies have shown that a period of physical activity has beneficial health effects,^16−18^ although these benefits may be offset by the impact of TRAP exposure on cardiopulmonary and lung function, with increases in inflammatory blood cells observed.^19−23^ However, the underlying biological mechanisms have not been fully elucidated, and classical epidemiological methods cannot accurately ascertain the short-term impacts of exposures to TRAP, largely due to the absence of robust and specific biomarkers. The advent of omics-based high-throughput technologies allows for molecular changes and biological pathways associated with TRAP exposure to be identified at a cellular level, and underlying pathomolecular mechanisms to be mapped more precisely.^24−27^ Metabolomics and transcriptomics provide powerful analytical methods to understand the molecular and biochemical pathways triggering a systemic response that can be observed in the peripheral blood.^28,29^ Metabolomics systematically investigates metabolites, such as amino acids, fatty acids, and lipids, and their impact on oxidative stress and inflammation pathways, in turn affecting human health and disease risk.^30,31^ Untargeted metabolomics offers a valuable approach to identifying and quantifying the effect of TRAP exposure on the blood metabolome.^28,32,33^ Transcriptomics allows the identification of gene expressions that are differentially induced and can be used as exposure biomarkers.^34^ Combining metabolomics and transcriptomics obtained from the same samples offers a more comprehensive approach to better understand the biological response and the pathways affected by exposure to TRAP, as it will extend the coverage of the molecules assayed and include a large variety of both endogenous and exogenous molecules. Integrating metabolomics and transcriptomics data contributes to systems biology by allowing for the construction of network models that represent the interactions between genes and metabolites. These network models can identify key nodes and edges that play crucial roles in maintaining the stability of biological systems, offering insights into how perturbations in these networks might lead to adverse health effects. In the present randomized crossover study, we characterize the associations between short-term exposure to traffic-related air pollution with metabolomic responses and genetic expression at a multiomic level among healthy participants and more vulnerable participants with COPD and ischemic heart disease (IHD). Our objective was to identify exposure-related dysregulated (multi-)OMIC patterns that may inform the mechanistic pathways affected by traffic-related air pollution.

## Materials and Methods

### Study Design

The experimental randomized, crossover study design exposed human subjects at the western end of Oxford Street, a busy Central London shopping street restricted to diesel-powered buses and taxicab traffic, and the nearby 142 ha (about 350 acres) traffic-free area of Hyde Park. The study has already been described elsewhere.^19^ In brief, the study population comprised a total of 120 volunteers, including healthy volunteers (*n* = 40), with no evidence of airflow obstruction, recruited through advertising placed within the public areas of the Royal Brompton Hospital, and volunteers with either COPD and no history of IHD (*n* = 40) or IHD (*n* = 40), with no evidence of airflow obstruction, recruited from the outpatient respiratory and cardiology clinics at the Royal Brompton & Harefield NHS Foundation Trust.^19^ One volunteer with IHD was excluded due to drop-out. All participants were required to have stopped smoking for at least 12 months. The study location for the first walk was randomized with the participants randomly assigned to walk for 2 h at one of the sites and 3–8 weeks later walk at the other site, being driven to and from each site in a hybrid car from the Royal Brompton Hospital. At each site, participants spent 2 h walking on predefined routes at their own pace from 11 am to 1 pm covering an average distance of 5 km, before being transported back to the hospital. The study design was highly standardized for exposures and blood sampling, thereby reducing the potential for technical confounding and allowing participants to act as their own control due to blood samples being taken before and after the experiments. Specifically, a blood sample was collected in each participant 2 h before, and 2 and 24 h after each walk. Participant information was collected to measure age, sex, health group, date of birth, body mass index (BMI), and blood pressure along with data for distance walked, diet, and medication.

Metabolomic analysis was performed on 60 volunteers. After the subjects with missing exposure measurements were excluded, our study population included a total of 50 volunteers (resulting in 300 metabolomic profiles). Details on exclusion criteria are illustrated in SI Figure S1. Transcriptomic analysis was based on the same blood samples, which were subsequently filtered based on exposure data availability and quality control checks (see below). Informed written consent was obtained from all participants, and the study was approved by the London City Road and Hampstead Ethics Committee.^19^

### Environmental Exposures

During each walk, TRAP exposures including PM_10_ and PM_2.5_, NO_2_, BC, and the total number of particles with a diameter less than 300 nm (PCNT), were measured in real time via a backpack each participant carried. It contained a light scattering sensor (AM510 SidePak Personal Aerosol Monitors, TSI Ltd., MI) to measure PM_10_ and PM_2.5_, a unipolar diffusion charger (Philips Aerosense NanoTracer; size range of 10–300 nm) to measure PCNT, a proxy for ultrafine particle concentrations, and a microAeth Model AE51 Black Carbon aerosol monitor (AEthlabs, CA; flow rate of 100 mL/min) for black carbon measurements. In Oxford Street, NO_2_ measurements were taken from a stationary kerbside monitoring site (51.51392, −0.15279) repeatedly passed during the Oxford Street walks. Due to the unavailability of monitoring sites in Hyde Park, NO_2_ measurements for the Hyde Park walks were taken from the nearest background monitoring site located in a school playground in North Kensington (51.52104, −0.21349). Temperature and relative humidity were electronically logged as were noise levels (Bruel and Kjaer Type 2236 Sound level meter, Naerum, Denmark).

### Omics Data Acquisition and Preprocessing

#### Metabolomics Data

The methods for the acquisition of untargeted metabolomics data have been described elsewhere.^31^ In brief, the analysis of serum samples was performed with an Agilent ultrahigh performance liquid chromatography coupled with a quadrupole time-of-flight spectrometer (UHPLC-QTOF). Separation was obtained with a reversed-phase column, ionization with and electrospray ionization in positive-ion mode. Feature finding was performed using Agilent Mass Hunter and Mass Profiler Pro software, as described earlier.

The raw metabolomic data set included *n* = metabolic features, from which *n* = 4027 were excluded following internal QC and due to missing measurements in 40% or more participants. Our final data set included *n* = 6040 metabolic features measured in 50 participants with full exposure data, which represented 300 metabolomic profiles (SI Figure S1). The data was log_2_ transformed and missing data were imputed using a quantile regression approach (impute.QRILC), implemented in the imputeLCMD R package,^35^ as described in previous studies,^31,36^ which has been shown to outperform other methods.^37^

The metabolic features identified in our analyses were categorized by their similarity in retention time and correlation in intensity across the samples to help identify ions potentially originating from the same compound. The different ions arising from the same metabolite were annotated whenever the ion species (e.g., different adducts or fragments) could be identified, based on their accurate mass and presence in the full-scan reference spectra of pure reference standards. Next, the *m*/*z* values were searched in the Human Metabolome Database (HMDB, www.hmdb.ca) using [M + H]^+^, [M–H_2_O + H]^+^, and [M + Na]^+^ as adducts and mass tolerance of ±8 ppm. For the selected main features, identifications were confirmed by comparison of retention times and tandem mass spectrometry (MS/MS) spectra between the features and pure reference standards when available. If standards were not accessible, the acquired MS/MS spectra were matched against those available in mzCloud (www.mzcloud.org). The level of confidence for the identification was based on the recommendations of the Chemical Analysis Working Group of the Metabolomics Standards Initiative (MSI) (SI Table 5).^38^

#### Transcriptomics Data

Preparation of RNA analysis for the Oxford Street II study was already described by Espín-Pérez et al.^25^ Briefly, the RiboPureTM-Blood kit (Ambion) was used to isolate total RNA from the blood samples extracted from participants after each exposure session (400 mL of whole blood and 1600 mL of RNA later) following the manufacturer’s instructions. The isolated RNA was hybridized on SurePrint G3 Human Gene Expression v2 or 3 8 × 60 K Microarray Kit using 200 ng of material. The Agilent Feature Extraction Software was used to extract raw data on the pixel intensities. Probes were matched to gene names based on their ID, using a database (accession ID GPL21185) on the NCBI platform. The resulting gene expression data set was cleaned for incorrectly labeled transcripts and log_2_ normalized resulting in 30,923 transcripts being assayed in 42 participants (252 profiles).

#### Correcting for Technical Confounding

Technical confounding introduced by samples being handled at different time and over multiple batches may induce additional (nuisance) variation,^39,40^ which, as previously proposed, can be corrected for by estimating a random effect for each technical confounder.^41,42^ Metabolomics analysis was conducted in 4 plates and 7 boxes and transcriptomics in 17 arrays and 12 batches, which were considered as technical confounders. This was corrected for by (i) fitting a linear mixed model for each omic measurement (with each metabolomic feature or transcript set as the dependent variable) setting the corresponding technical confounders as random intercepts (plate and box for metabolomics data, and array and batch for transcriptomics data) and (ii) subtracting the corresponding random effect estimates from the measured level of each (metabolomic or transcriptomic) biomarker. The resulting denoised data were used for subsequent statistical modeling.

### Statistical Analysis

#### Association Study

A principal component analysis (PCA) was performed on the metabolomics data for all 300 samples. Five outlying samples, each from a different individual, were removed and the remaining 295 samples were used for subsequent regression analysis (SI Figure S2).

To accommodate repeated measurements in the study, a flexible multivariate normal (MVN) model was fitted to identify metabolic^31^ and transcriptomic^25^ features associated with exposure to TRAPs, using the gls function from the nlme package.^43^ Each metabolic feature/mRNA (*Y*) was modeled to follow a multivariate normal distribution with a mean vector μ and a covariance matrix ∑, using the equation:
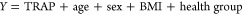
where *Y* ∼ MVN(μ,∑). MVN regression explicitly models the within-individual variability, and hence individuals act as their own controls and implicitly correct for individual characteristics. Nevertheless, to account for potential residual confounding, we have included age (continuous), sex (female or male), and health status (categorical variable with 3 levels Healthy, IHD, or COPD) as fixed effects in our models. Temperature and humidity were not associated with TRAP exposure in our data and were therefore not considered as potential confounders in our model. An unspecified variance–covariance matrix was modeled, using the CorSymm function, where each subject ID was used as a grouping factor, with a constant variance for each combination of time point (2 h before, and 2 and 24 h after each walk) and site (Oxford Street or Hyde Park), using the varIdent function.^43^

In order to account for the correlation and possible (partial) redundancies across omic features and prevent too-stringent corrections for multiple testing, we defined our per-test significance level using a Bonferroni correction for the effective number of tests (ENT) performed, ensuring a family-wise error rate below 0.05. As an alternative to permutation-based calculation of ENT,^44,45^ we defined here the ENT as the number of principal components needed to explain >99% of variance of the full data.^46^ For consistency, this correction was also performed for metabolomics data (ENT = 284 and 202 for metabolomics and transcriptomics data, respectively). Small sample size in each of the health status groups (*N* = 18 Healthy, *N* = 18 COPD, and *N* = 14 IHD) prevented us from running stratified analyses by health groups.

#### Pathway Analyses

The Mummichog (v2.0) algorithm was used to perform functional analysis of metabolic features associated with TRAP,^47^ using the “functional analysis” on MetaboAnalyst (https://www.metaboanalyst.ca/MetaboAnalyst/ModuleView.xhtml).^48^ The *p*-values and *t*-scores for the output of each MVN model for each TRAP were used as the input for the Mummichog algorithm to perform an over-representation analysis and calculate an enrichment *p*-value using a Fisher exact test, allowing a mass tolerance of 8.0 ppm in positive mode. The default database provided on Metaboanalyst was used, which combined KEGG, BiGG, and the Edinburgh Model. We used the significant thresholds inferred within Metaboanalyst, which was 0.05 for PM_2.5_ and PM_10_, 0.01 for PCNT and BC, and 0.005 for NO_2_.

#### Conditional Independence Network

We adopted a conditional independence network approach to visualize the complex (partial) correlation structure across TRAP-associated metabolic features. We used a graphical LASSO (gLASSO) model calibrated via stability as implemented in the sharp package.^49^ In brief, gLASSO was applied to (*n* = 500) 80% subsamples of the study population and for different values of the penalty parameter (controlling the sparsity of the graph). For each value of the penalty parameter, edge selection proportion was calculated as the number of times the edge was included across the 500 subsamples and the two hyper-parameters: the penalty and the threshold in selection proportion (controlling the stability of the model) were calibrated jointly so they maximized a likelihood-based stability score.^49^

In order to aid visualization of the network, the metabolic features found associated with TRAP were summarized using stability-calibrated consensus clusterin.^50^ The optimal number of clusters was calibrated using (*n* = 500) 80% subsamples, maximizing comembership counts across subsamples. Each of the resulting clusters was represented by its medoid.

This analysis was extended to also include TRAP-associated mRNA. The resulting multiomic network was also calibrated via stability, but block-specific hyper-parameters were considered.^49^ For clarity, we only included TRAP-associated mRNA that were mapped to a known gene.

Metabolomic and multiomic networks were estimated separately at three time points: 2 h before, and 2 and 24 h after each walk.

## Results and Discussion

### Data Overview

Our study population included 18 healthy volunteers, 18 volunteers with COPD, and 14 volunteers with IHD. Their characteristics are reported in Supporting Table 1. There were overall more male (*n* = 32) than female (*n* = 18) participants, especially in the IHD group. The mean age of participants was 65.5 years and was similar across the three groups. BMI, diastolic, and systolic blood pressures were also similar in the three groups.

All five TRAP exposures were significantly higher in Oxford Street than in Hyde Park (SI Table S2). Although PM_10_, PM_2.5_, and NO_2_ concentrations in Oxford Street and Hyde Park showed some overlaps in their interquartile ranges, BC and PCNT had the most significant differences with no overlap (SI Figure S3 and Table S2).

### Omics Markers of TRAP Exposures

**Figure 1:**
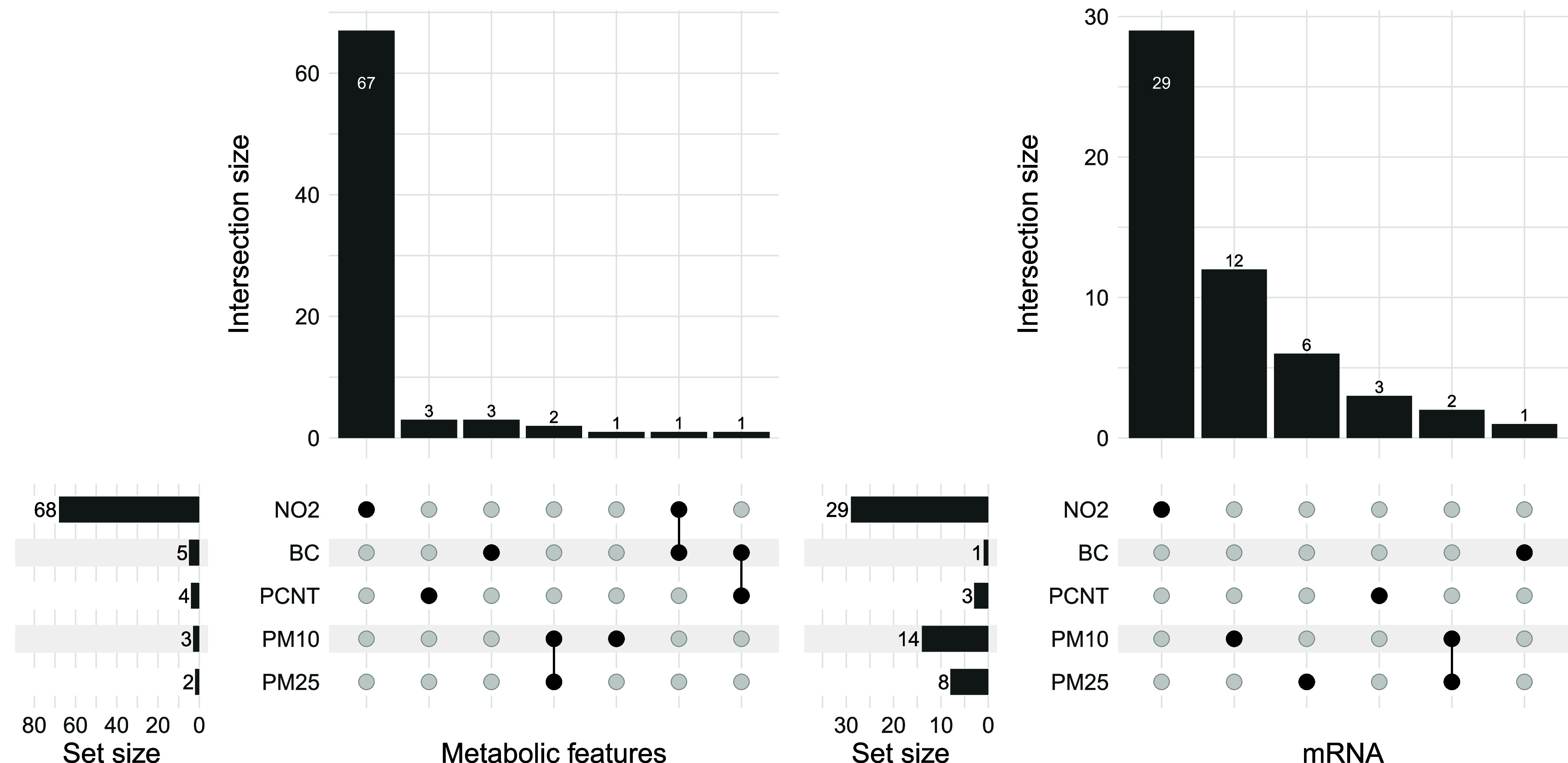
Upset plots indicating the number of unique metabolic features (A) and mRNA (B) significantly associated with at least one TRAP exposure in Oxford Street. (A) Illustration of the number of metabolic features associated with each TRAP or combination of TRAPs with our MVN and after Bonferroni correction. (B) Summary of the total number of significant mRNA associated with each TRAP with our MVN and after Bonferroni correction using the ENT (*n* = 202).

In our univariate analysis, each metabolic feature was regressed against each TRAP exposure, adjusting for age, sex, BMI, and health group. Under a Bonferroni correction for ENT = 284 tests, we identified 230 unique metabolic features significantly associated with at least one TRAP exposure. Of these, 168 were associated with NO_2_, 43 with BC, 18 with PCNT, 14 with PM_10_, and 8 with PM_2.5_ (SI Figure S4). As a more conservative alternative, we corrected for multiple testing using a Bonferroni correction for *n* = 6040 tests (i.e., ignoring the correlation across metabolic features), the total number of assayed features after QC filtering. This identified a unique set of 78 metabolic features associated with at least one TRAP exposure (Figure 1A and SI Figure S4).

**Figure 2:**
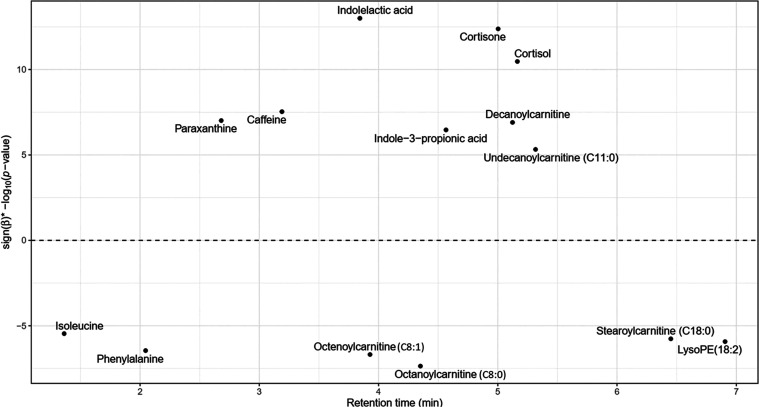
Manhattan plot illustrating the association between annotated metabolites and exposure to NO_2_ in our univariate analysis. The −log_10_ transformed *p*-values, multiplied by their direction of association (sign of the β coefficients) for each metabolite, were plotted against their retention times in minutes. The 14 metabolites shown were annotated at MSI levels 1 and 2 and were significantly associated with NO_2_ exposure after Bonferroni correction for *n* = 6040 tests. For compounds where associated metabolic features were also significant, only the main metabolic features were shown here (see SI Table S5 for more details on annotation).

The dominant pollutant exposure was NO_2_ accounting for 67 unique metabolite associations (Figures 1A and 2).

PCNT, BC, and PM_10_ exposure showed 3, 3, and 1 exclusive associations, respectively. There was no significant feature exclusively linked to exposure to PM_2.5_. Two additional features showed common associations with PM_10_ and PM_2.5_, and two additional features with common associations with NO_2_ and BC, and BC and PCNT, respectively (Figure 1A). Consensus clustering on these 78 features identified 63 clusters (SI Figure S5), 52 of which included a single feature, 8 included 2 features, 2 included 3 features, and 1 included four features (SI Figure S6). All TRAP-associated features, except one feature from cluster 56, had retention times similar to those of the other features belonging to the same cluster (irrespective of their association with TRAP exposure), potentially indicating their structural proximity.

Our analysis of mRNA after Bonferroni correction for ENT = 202 tests identified 53 unique mRNAs associated with at least one TRAP exposure (Figure 1B and SI Figure S7). Of these, 29 were associated exclusively with exposure to NO_2_ (Figure 1B). A further 14 and 8 mRNAs were associated with PM_10_ or PM_2.5_, respectively, and 3 mRNAs were uniquely associated with exposure to PCNT and one to BC. Two mRNAs were associated with exposure to both PM_10_ and PM_2.5_. Of these 53 TRAP-related mRNAs, 38 were mapped to a known gene (SI Table 3).

Our results partially differed from the original work carried out on this data.^25^ We previously identified 29 metabolic features significantly associated with TRAP exposure, including 26 related to NO_2_ exposure. Of these 18 NO_2_-associated features overlapped with our current findings. Both studies identified a small number of associations with PM_10_ and BC and no unique associations with PM_2.5_: in both studies, associations to PM_2.5_ were only found in conjunction with PM_10_. Minimal overlap of significant metabolic features was observed between TRAP exposures, suggesting that each pollutant could potentially exert its own effect, affecting specific molecular pathways.

Significantly fewer mRNAs were identified as associated with TRAPs here with respect to Espin-Perez et al.^44,50^ These differences can be explained by our refined data preprocessing, which is a key step to obtaining sound and solid results.^44,51^ Additionally, for both omics, the data quality was strongly improved by identifying and removing five outlying observations (SI Figure S2). The differing approaches to data cleansing and exposure data have led to a greater number of metabolite features associated with TRAP exposure in this study and a lower number of mRNAs. However, in both approaches, NO_2_ was found to be the dominant pollutant and there was minimal overlap between metabolomic markers of TRAP exposure, possibly suggesting exposure-specific metabolic pathways.

### Pathway Enrichment and Annotations

**Figure 3:**
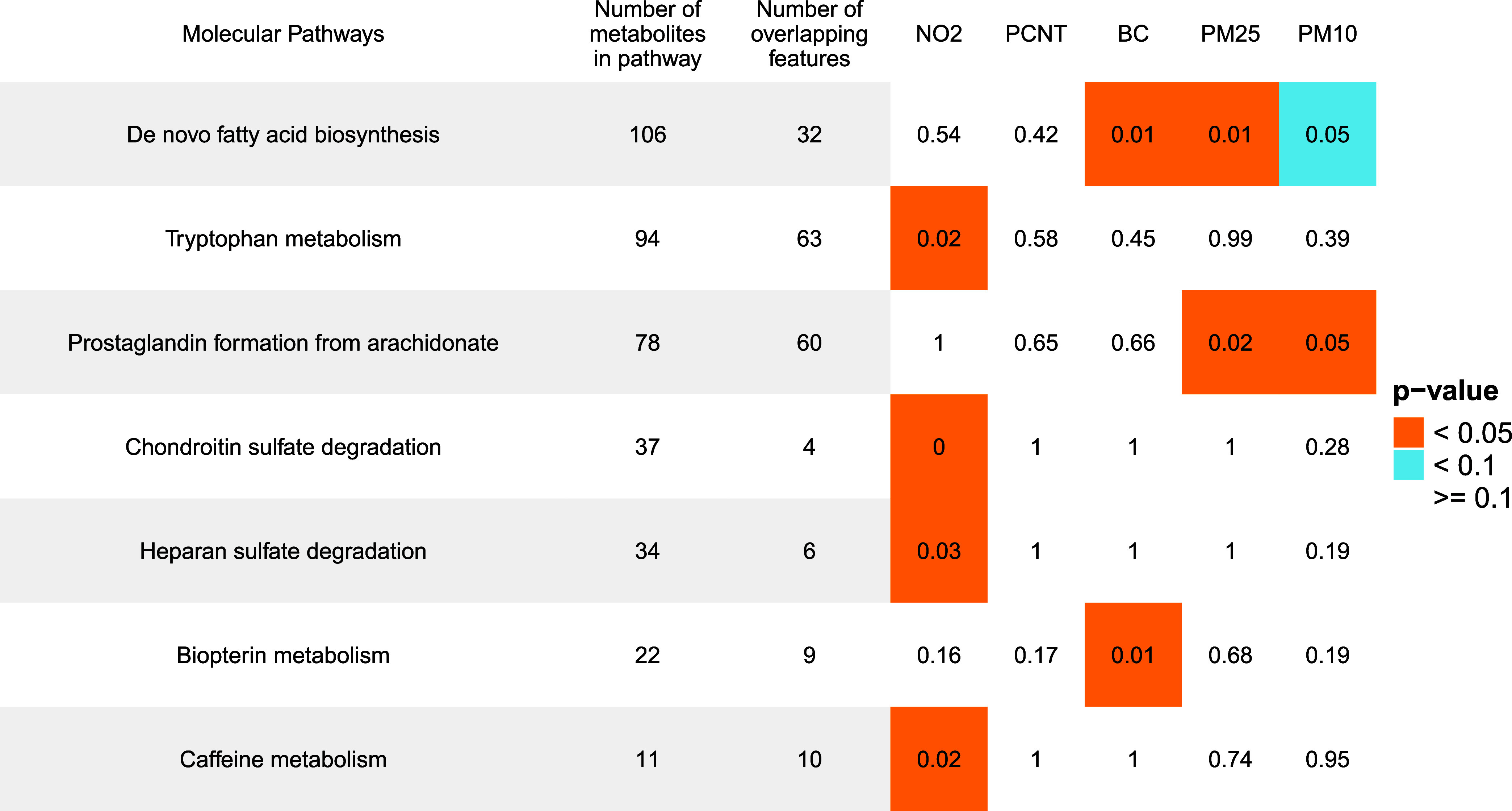
Functional analysis indicating the pathways associated with each TRAP exposure. Colors indicate the strength of the association (orange for *p*-value <0.05 and blue for *p*-value <0.1). We report the number of features identified by the mummichog in our data set for each pathway (column 3) and the number of features included in the pathway as part of the mummichog database (column 2).

We applied the Mummichog algorithm,^47^ using the full list of (*n* = 6040) *p*-values and t-scores from the MVN models for each TRAP exposure as the input. We identified 7 unique enriched pathways with a significance level of *p* < 0.05 (Figure 3).

All pathways included at least 4 putative compound matches. One pathway was identified as associated with both PM_2.5_ and PM_10_ exposure (prostaglandin formation) and one to PM_2.5_, PM_10_, and BC (de novo fatty acid biosynthesis). Four pathways were exclusively associated with NO_2_ exposure (tryptophan metabolism, chondroitin sulfate degradation, heparan sulfate degradation, and caffeine metabolism). Biopterin metabolism was associated with BC exposure, but not other pollutants.

We performed detailed annotations on our selected features to further verify these enriched pathways. Out of the 78 features associated with TRAP, 21 could be annotated (MSI level 1 or 2) (Figure 2 and SI Table 4), corresponding to a total of 14 unique metabolites.^31^ All of the annotated features were associated with NO_2_ exposure. Of these 14 compounds, 4 compounds (caffeine, phenylalanine, 2 acyl-carnitines), were also found in our previous work.^33^ Our new analysis revealed higher levels of cortisol and cortisone, indoleactic acid (IAA), and indole-3-propionic acid (IPA) associated with TRAP exposure, as well as lower levels of isoleucine (SI Table 4). These annotations confirmed 3 pathways identified by Mummichog: that of the Biopterin metabolism (phenylalanine), of the caffeine metabolism (Caffeine, Paraxanthine), and of the tryptophan metabolism (IAA, IPA). Our pathway analysis points to metabolic alterations, more specifically to oxidative stress perturbations, which were previously associated with exposures to TRAP.^51^

Tryptophan is an essential amino acid for protein synthesis that processes such as gastrointestinal functions, immunity, metabolism, and the nervous system. It can be degraded through three main pathways: kynurenine, serotonin, and indole pathway. The last one involves the gut microbiome which, through enzymatic reactions, can degrade tryptophan into downstream metabolites such as IAA and IPA. Our data indicate that short-term air pollution exposure to NO_2_ in TRAP significantly affects the indole pathway with increasing levels of IAA and IPA.

To explore this hypothesis further, we mined our metabolomic data for tryptophan and kynurenine, which are key to tryptophan metabolism. An imbalance of the kynurenine/tryptophan ratio has appeared in a study to be associated with other diseases.^52^ Both compounds were identified in our data when searching for their accurate mass and known retention times. Tryptophan was found positively associated with NO_2_ exposure (*p* = 0.0000305, β = 0.0312) and kynurenine negatively associated (*p* = 0.00417, β = −0.0024), indicating an imbalance between tryptophan and kynurenine associated with NO_2_ exposure. Emerging evidence indicates that air pollutants could potentially influence the human microbiome (gut, lung, and skin), as recently reviewed by Mousavi et al.^53^ Alterations in gut microbiome composition associated with TRAP exposure have been observed in normal and overweight adolescents^54,55^ and asthmatic children,^56^ although specific mechanisms have not been well characterized in humans. Here, we also observe increased levels of cortisone and cortisol with NO_2_ exposure. Interestingly, elevated levels of glucocorticoids have been observed in TRAP-exposed populations in China in two recent studies.^57,58^ Associations were described here for exposure to PM although NO_2_ exposure was not measured. Several studies suggest a potential link between exposure to TRAP and the activation of the hypothalamic–pituitary–adrenal axis (HPA) realizing glucocorticoids and inducing gut microbiome dysbiosis^57,59,60^ which is in accordance with our observations.

### Network Analysis

#### Metabolomics Network Analysis

**Figure 4:**
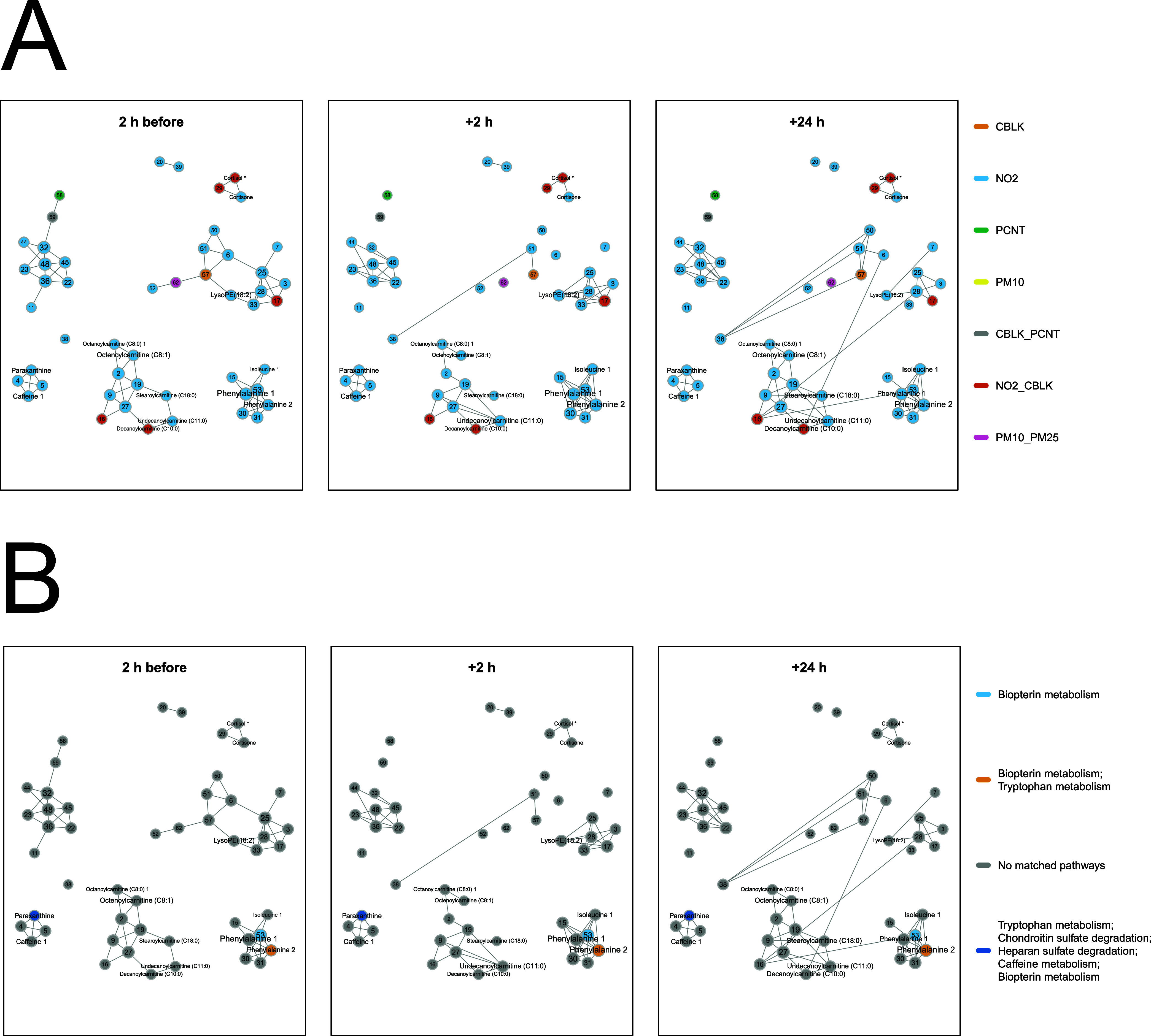
Conditional independence network on clusters of metabolic features significantly associated with TRAP exposure, at 2 h before visits, and 2 and 24 h after visits. Each node represents a feature or cluster of features and each edge represents a correlation between two features conditional on all other features. Clusters that contain the main feature for the corresponding compound are labeled with asterisks. (A) Nodes are colored by association with each TRAP exposure. (B) Nodes are colored by association with one or multiple pathways enriched with the mummichog tool (see SI Table S5 for more details on annotation).

Conditional independence network models were applied to the 63 metabolic clusters significantly associated with at least one TRAP at each time point (Figure 4) and were calibrated via stability (SI Figure S8).

Metabolic clusters were colored by their association with a TRAP (Figure 4A) or according to their implication in a Mummichog pathway (Figure 4B). The network inferred on data measured 2 h before each walk may be interpreted as the individual metabolomic status (restricted to TRAP-related features) before (differential) exposures. Topological comparison of networks inferred before and 2 or 24 h after the walks could inform on acute and lagged exposure-related changes in the metabolic profiles after exposure. Overall, the baseline network shows that metabolic clusters associated with each TRAP are not necessarily linked (Figure 4A) and that some pathways may be shared between TRAP-related metabolic features (Figure 4B). The topology of the network inferred 2 h after exposure is overall similar to that of the baseline network, suggesting modest or local modifications in the correlation structure across metabolic features. However, several changes, in particular, in modules including LysoPE were observed. These changes may indicate pathway modifications and alterations associated with the exposure. In the network inferred 24 h after exposure, some rewiring is observed and some of the correlation patterns revert to those estimated before the experiment. Some partial correlations (e.g., those involving the Lyso PE module) remain altered 24 h after the exposure, which may suggest persistent effects of TRAP exposures on the metabolome. Some edges appeared in the network estimated 24 h after exposures (e.g., edges involving cluster 38), which may be indicating some lagging effects of TRAP exposures.

**Figure 5:**
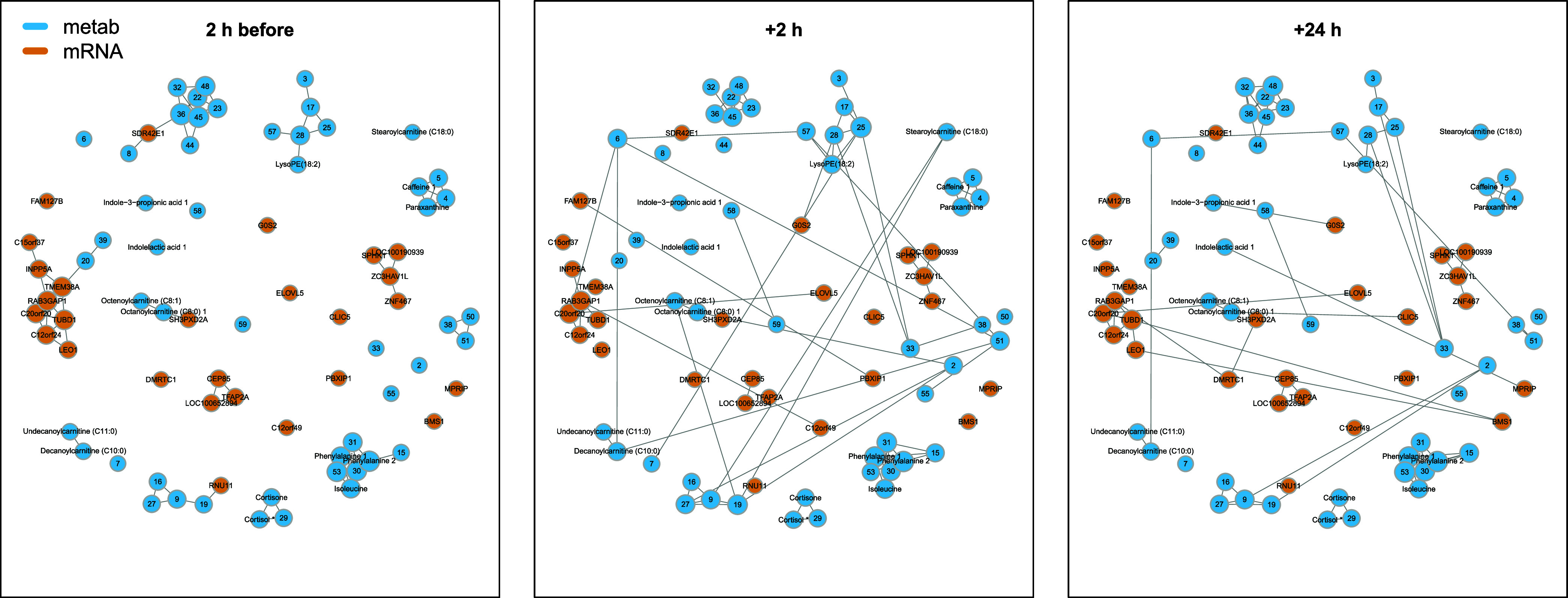
Conditional independence network on 63 clusters of metabolic features and 38 mRNA significantly associated with TRAP exposure, at 2 h before visits, 2 and 24 h after visits. Each node represents a feature or cluster of features and each edge represents a correlation between two features conditional on all other features. Clusters that contain the main feature for the corresponding compound are labeled with asterisks (see SI Table S5 for more details on annotation).

Unlike previous studies, where molecular data were analyzed in isolation, we were able here to integrate both transcriptomic and metabolomic data to investigate the correlation structures driving the variance–covariance between all TRAP-related features we identified. Specifically, we estimated multiomic conditional independence networks combining the 63 metabolic clusters and the 38 mRNA with gene symbols (of the 53 TRAP-related mRNA). As before, the network was estimated 2 h before and 2 and 24 h after each walk and calibrated via stability (see calibration plots in SI Figure S9). In the baseline network, we identified four cross-omic edges, between cluster 8 and SDR42E1, cluster 20 and TMEM38A, cluster 19 and RNU11, and Octanoylcarnitine (cluster 38) and SR3PXD2A (Figure 5).

Two hours after exposure, the topology of the network has been substantially modified and, in particular, these cross-omics edges disappear. However, some new cross-omic links between clusters 17 and 57 and GOS2, between cluster 6 and GOS2 and TMEM38A, and between Octanoylcarnitine and DMRTC1 were estimated. Two additional interomics edges appear 24 h after exposure, IPA and cluster 6 and 57 with GOS2 edge Undecanoylcarnitine with MPRIP. No specific function was found associated with these mRNAs, except for GOS2 which is involved in multiple pathways of metabolism regulation, including lipid metabolism.^61^ Its association with IPA and carnitines in our network could potentially point to a perturbation in lipid metabolism associated with gut permeability and microbiome activity.

With our conditional networks, we could visualize that the same metabolites were involved in multiple pathways, indicating complex and intricate mechanisms associated with TRAP exposures. We also observed differential topologies 2 h following exposure for the leukotriene and linoleate pathway, which did not revert to their initial state 24 h after exposure, potentially suggesting an acute and longer-term effect of TRAP exposure.

Exposure to particulate matter shared no common pathways with other exposures, potentially indicating different effects for these types of TRAP. Overall, most of the described pathways associated with TRAP in our study were related to increased oxidative stress and inflammation, in accordance with what is observed in the literature for air pollution.

We identified NO_2_ as the predominant TRAP species with the most associations with both metabolic features and mRNA. Notably, our investigation strongly supports the hypothesis of a connection between NO_2_ exposure and the equilibrium of tryptophan and kynurenine, underscoring the influence of air pollutants on the human microbiome.

Through adapting novel statistical approaches, this work integrates transcriptomics and metabolomics on the same participants across multiple time points and sheds new light on potential multiomic alterations associated with TRAP exposure. This analytical approach can also be readily extended to similar exposomic studies.

There are several limitations to this study. First, NO_2_ was not directly measured but was obtained from the nearest monitoring station. While this may result in a lower granularity in the exposure data, which may not fully reflect the individual-level NO_2_ exposure during the walk, we expect that this may not have an impact on our data as walks in both locations were standardized. In addition, it is expected that data from these monitoring stations are more accurate and reliable than those from smaller sensors measuring NO_2_. In terms of generalizability, a major source of TRAP exposures was diesel vehicles. As such, different effects could have been found in a study where there were more petrol vehicles. The population examined had an average age of 65, and consequently, the conclusions may be less applicable to a younger population. In addition, we could not make inference on the effects of TRAP on the metabolome/transcriptome in specific health groups (healthy, COPD, and IHD), as the small sample sizes were within each health group, limiting the power to perform stratified analyses. Finally, the relatively small sample size of 50 participants and variations in data processing and exposure concentrations compared to prior research can introduce variability in the identified metabolites and pathways associated with TRAP exposure. Standardizing data processing and cleansing methods across studies could enhance comparability and robustness.

Despite the limitations, in this comprehensive crossover study conducted at two distinct sites, encompassing healthy individuals and those with COPD and IHD, we have revealed many metabolic and mRNA modifications as well as their interactions, linked to high and low TRAP exposure. Our findings offer compelling evidence of a potential impact on gut microbiome dysbiosis due to short-term NO_2_ exposure.

#### Declaration of Competing Financial Interests

MC-H holds shares in the O-SMOSE company and has no conflict of interest to disclose. Consulting activities conducted by the company are independent of the present work. The authors declare no conflict of interest to disclose. Where authors are identified as personnel of the International Agency for Research on Cancer/World Health Organization, the authors alone are responsible for the views expressed in this article, and they do not necessarily represent the decisions, policy, or views of the International Agency for Research on Cancer/World Health Organization.

## Supporting Information Available

Detailed information on the study population, exposure measurements, and both the transcripts and metabolic features found associated with TRAP exposure, details on our analytical plan, the descriptive analysis of our metabolomic and exposure data, and full resolution results of our association studies along with a detailed description of the metabolic features found associated with TRAP exposures and visualizations of the calibration procedure for both our clustering and network models (PDF)
