# Correction to “Multiomic Signatures of Traffic-Related Air Pollution in London Reveal Potential Short-Term Perturbations in Gut Microbiome-Related Pathways”

**DOI:** 10.1021/acs.est.4c09132

**Published:** 2024-09-16

**Authors:** Sibo Lucas Cheng, Michael Hedges, Pekka Keski-Rahkonen, Anastasia Chrysovalantou Chatziioannou, Augustin Scalbert, Kian Fan Chung, Rudy Sinharay, David C. Green, Theo M. C. M. de Kok, Jelle Vlaanderen, Soterios A. Kyrtopoulos, Frank Kelly, Lützen Portengen, Paolo Vineis, Roel C. H. Vermeulen, Marc Chadeau-Hyam, Sonia Dagnino

**Affiliations:** † MRC Centre for Environment and Health, Department of Epidemiology and Biostatistics, School of Public Health, Imperial College London, London, W12 7TA, United Kingdom; ‡ MRC Centre for Environment and Health, Environmental Research Group, Imperial College London, London, W12 0BZ, United Kingdom; § International Agency for Research on Cancer (IARC), Lyon, 69366 Cedex, France; ⊥ National Heart & Lung Institute, Imperial College London, London, SW7 2AZ, United Kingdom; ¶ Royal Brompton & Harefield NHS Trust, London, SW3 6NP, United Kingdom; □ Imperial College Healthcare NHS Trust, London, W2 1NY, United Kingdom; ■ Department of Toxicogenomics, GROW School for Oncology and Reproduction, Maastricht University, Maastricht, 6229 ER, the Netherlands; ○ Division of Environmental Epidemiology, Institute for Risk Assessment Sciences, Utrecht University, Utrecht, 3584 CS, the Netherlands; ● National Hellenic Research Foundation, Athens, 11635, Greece; △ Julius Centre for Health Sciences and Primary Care, University Medical Centre, Utrecht University, Utrecht, 3584 CG, the Netherlands; ▲ Transporters in Imaging and Radiotherapy in Oncology (TIRO), School of Medicine, Direction de la Recherche Fondamentale (DRF), Institut des sciences du vivant Fréderic Joliot, Commissariat à l’Energie Atomique et aux énergies alternatives (CEA), Université Côte d’Azur (UniCA), Nice, 06107, France

The Acknowledgments and the authors’ affiliation have been corrected.

