# Supplementary material for: Multiomic Signatures of Traffic-Related Air Pollution in London Reveal Potential Short-Term Perturbations in Gut Microbiome-Related Pathways
Source: Environ Sci Technol. 2024 May 10;58(20):8771–82. doi: 10.1021/acs.est.3c09148 (PMC11112755; doi:10.1021/acs.est.3c09148)
Supplement: Supplementary file 1 — es3c09148_si_001.pdf [file es3c09148_si_001.pdf]

Supplementary Materials:

Multi-omic signatures of traffic related air pollution in London reveal potential short-term perturbations in gut microbiome-related pathways

Sibo Lucas Cheng[1,2,+] Michael Hedges[3,+] Pekka Keski-Rahkonen[4], Anastasia Chrysovalantou Chatziioannou[4], Augustin Scalbert[4], Kian Fan Chung[5,6], Rudy Sinharay[5,7], David C Green[1,3], Theo M.C.M. de Kok[8], Jelle Vlaanderen[9], Soterios A Kyrtopoulos[10], Frank Kelly[1,3], Lützen Portengen[9], Paolo Vineis[2], Roel CH Vermeulen[9,11], Marc Chadeau-Hyam[1,2,#*], Sonia Dagnino[2,12,#*]

[1] NIHR HPRU in Environmental Exposures and Health, Imperial College London, London, W12 0BZ, United Kingdom
[2] MRC Centre for Environment and Health, Department of Epidemiology and Biostatistics, School of Public Health, Imperial College London, London, W12 7TA, United Kingdom
[3] MRC Centre for Environment and Health, Environmental Research Group, Imperial College London, London, W12 0BZ, United Kingdom
[4] International Agency for Research on Cancer (IARC), Lyon, 69366 Cedex, France
[5] National Heart & Lung Institute, Imperial College London, London, SW7 2AZ, United Kingdom
[6] Royal Brompton & Harefield NHS Trust, London, SW3 6NP, United Kingdom
[7] Imperial College Healthcare NHS Trust, London, W2 1NY, United Kingdom
[8] Department of Toxicogenomics, GROW School for Oncology and Reproduction, Maastricht University, Maastricht, 6229 ER, the Netherlands
[9] Division of Environmental Epidemiology, Institute for Risk Assessment Sciences, Utrecht University, Utrecht, 3584 CS, the Netherlands
[10] National Hellenic Research Foundation, Athens, 11635, Greece
[11] Julius Centre for Health Sciences and Primary Care, University Medical Centre, Utrecht University, Utrecht, 3584 CG, the Netherlands
[12] Transporters in Imaging and Radiotherapy in Oncology (TIRO), School of Medicine, Direction de la Recherche Fondamentale (DRF), Institut des sciences du vivant Fréderic Joliot, Commissariat à l'Energie Atomique et aux énergies alternatives (CEA), Université Côte d'Azur (UniCA), Nice, 06107, France

[+] Joint first authors
[#] Joint last authors

Corresponding authors
*Emails : sonia.dagnino@univ-cotedazur.fr , m.chadeau@imperial.ac.uk

Summary content:

- 20 pages
- 5 Tables
- 9 Figures

Table of Contents

SI Table 1. Characteristics of the study population.

SI Table 2. Description of measured exposures.

SI Table 3. List of mRNAs significantly associated with at least one TRAP with existing gene names.

SI Table 4. List of metabolic features significantly associated with at least one TRAP, their cluster membership, and annotation.

SI Table 5. Annotation of metabolic features.

SI Figure S1. Schematic representation of the study population and exclusion criteria.

SI Figure S2. Score plots of the first (x-axis) and second (y-axis) principal components from principal component analysis (PCA) of 300 metabolic profiles of the 50 subjects.

SI Figure S3. Boxplots showing distributions of measured exposures in Oxford Street and Hyde Park.

SI Figure S4. Manhattan plots showing the -log10 transformed p-values of metabolic features plotted against their retention time for each TRAP exposure.

SI Figure S5. Calibration plot for consensus clustering of metabolomics.

SI Figure S6. Scatter plot of molecular mass of 78 metabolic features plotted against their retention time (min).

SI Figure S7. Volcano plots for transcriptomics.

SI Figure S8. Calibration plots for metabolomics networks.

SI Figure S9. Calibration plots for multi-omic networks.

SI Table 1: Characteristics of the study population with full exposure data in the Oxford Street II study.

COPD: Chronic obstructive pulmonary disease, IHD: ischaemic heart disease. *P*-values from paired t-tests for continuous variables and chi-square tests for categorical variables were shown.

	COPD (n = 18)	Healthy (n = 18)	IHD (n = 14)	Overall	p-value
Sex					
Female	8 (44.4%)	9 (50.0%)	1 (7.1%)	18 (36.0%)	0.028
Male	10 (55.6%)	9 (50.0%)	13 (92.9%)	32 (64.0%)	
Age					
Mean (sd)	67.7 (5.71)	64.1 (6.77)	64.5 (6.45)	65.5 (6.41)	0.661
Body Mass Index					
Mean (sd)	25.6 (3.88)	21.4 (3.41)	27.6 (4.28)	24.6 (4.58)	0.446
Diastolic Blood Pressure (mmHg)					
Mean (sd)	152 (17.0)	136 (18.3)	126 (14.2)	137 (19.0)	0.26
Systolic Blood Pressure (mmHg)					
Mean (sd)	90.0 (7.62)	74.7 (9.08)	76.0 (9.02)	79.0 (10.6)	0.468

SI Table 2: Description of exposures in the Oxford Street II study.

BC: Black carbon, PCNT: Total number of particles. *P*-values were calculated using paired t-test.

	Hyde Park (n=50)			Oxford St (n=50)			p-value
	N	Mean (sd)	Range	N	Mean (sd)	Range	
PM_{10} (µg m^{-3})	50	23 (16.4)	3.7-80.2	49	32.4 (14.7)	14.1-84.4	0.00105
$PM_{2.5}$ (µg m^{-3})	50	11.2 (13.0)	3.36-60.8	49	20.5 (12.9)	6.4-75.5	<0.001
NO_2 (µg m^{-3})	41	10.3 (7.71)	3.01-40.5	38	17.3 (7.56)	6.1-46.2	<0.001
BC (µg m^{-3})	47	1.75 (1.24)	0.29-5.16	49	10.9 (3.12)	6.73-17.8	<0.001
PCNT (particles # cm^{-3})	50	6738 (3269)	2846-15687	48	24836 (8088)	7197-38638	<0.001
Noise (Db)	46	73.3 (3.83)	61.4-78.0	43	76.2 (1.96)	71.4-81.2	<0.001
Relative Humidity (%)	49	53.4 (13.4)	27.9-87.4	48	47.5 (18.1)	9.37-81.0	0.0689
Temperature (OC)	49	17.0 (6.70)	2.63-27.2	48	20.2 (8.05)	2.34-31.4	0.0152

SI Table 3: mRNAs significantly associated to TRAP with existing gene symbols.

List of all 38 mRNA that were found as significantly associated to at least one TRAP in our MVN model with their corresponding gene symbol and Beta coefficients.

		Regression coefficients				
Accession number	Gene symbol	PM25	PM10	NO2	BC	PCNT
A_24_P373844	KCTD15	0.00165777	0.0016477	**0.01647895**	0.01309058	6.16E-06
A_23_P35456	SH3PXD2A	-0.0016961	0.00078837	**-0.0056235**	0.00154447	-1.54E-07
A_23_P129458	SDR42E1	-0.0028878	-0.0002495	**-0.0084621**	-0.0054769	-8.73E-07
A_23_P74609	G0S2	0.00447517	-0.0011805	**0.02123222**	-0.0093609	-1.65E-06
A_33_P3378056	TFAP2A	-0.0018742	-0.0018	**0.01657733**	0.00763885	6.88E-06
A_21_P0014020	LOC100652894	-0.0009254	-0.0021256	**0.01346835**	0.00075634	1.40E-06
A_33_P3214432	ZC3HAV1L	-0.0006607	0.00748795	**-0.0219629**	0.02582521	9.29E-06
A_23_P200874	CEP85	0.00216714	0.00065595	**0.01147049**	0.0030797	2.35E-06
A_33_P3238052	C12orf24	-0.0024834	-0.0013697	**-0.0056377**	-0.0054273	-3.84E-06
A_33_P3222069	SPHK1	0.00797911	0.01329894	**-0.0172166**	0.01971206	1.21E-05
A_24_P110914	LOC100190939	0.00032535	0.00667395	**-0.0236742**	0.0387678	1.55E-05
A_23_P50942	RAB3GAP1	-0.0024316	-0.0012112	**-0.0036613**	-0.0022998	-1.15E-06
A_23_P160618	SH2D2A	0.00046065	-0.0004135	**0.00667315**	-0.0090209	-4.73E-06
A_24_P26897	INPP5A	-0.0040903	-0.0019997	**-0.0062311**	-0.0054748	-5.29E-06
A_21_P0014093	LOC100507039	-0.0057799	0.00071668	**-0.0114499**	0.02211534	8.37E-06
A_32_P179998	DMRTC1	-0.0070384	-0.0057513	**-0.007327**	-0.0165631	-8.10E-06
A_24_P218074	ZNF467	-0.0070019	-0.0053192	**-0.0099231**	0.0069592	-3.61E-07
A_23_P17307	C20orf20	-0.002697	-0.0026592	**-0.0042223**	-0.0024362	-2.60E-06
A_23_P416774	CLIC5	0.00515385	0.00436823	0.00145155	0.01327942	**7.33E-06**
A_33_P3273719	ELOVL5	-0.0028675	-0.0019266	2.42E-05	-0.0115789	**-7.55E-06**
A_21_P0000664	CD4	-0.0073369	-0.005244	-0.0074411	-0.019483	**-1.48E-05**
A_21_P0000502	RNU11	-0.006583	**-0.0091321**	-0.0038552	-0.0155414	-4.85E-06
A_24_P18105	ASPH	-0.0085671	**-0.0099755**	-0.0003802	-0.0067193	-6.23E-06
A_19_P00803575	MPRIP	0.01227539	**0.01242791**	-0.0015842	0.01857774	8.11E-06
A_23_P143143	ID2	-0.0054379	**-0.005371**	0.000733	-0.0038134	-2.45E-06
A_33_P3270109	LEO1	-0.0061374	**-0.006666**	-0.0015051	-0.0035368	-4.24E-07
A_21_P0000508	SNAR-D	-0.0084485	**-0.0091237**	-0.0037879	-0.0031301	-2.53E-06
A_24_P226355	RANBP9	-0.0071246	**-0.0074181**	-0.0012348	-0.0052291	-1.35E-06
A_33_P3410011	PBXIP1	-0.011505	**-0.0128731**	-0.0048778	-0.0090772	-4.50E-06
A_33_P3296169	CDCA7	-0.0070674	**-0.0079957**	-0.0030621	-0.0153971	-7.41E-06
A_24_P109633	ITPK1	0.00637306	**0.00800379**	0.00188442	0.00584336	7.97E-07
A_23_P101392	TMEM38A	**-0.0067507**	-0.0052426	-0.0045572	-0.0081761	-4.94E-06
A_33_P3239134	POM121L4P	**-0.0099143**	-0.0052324	0.00020308	0.00524576	1.39E-06
A_32_P85330	C15orf37	**-0.011681**	-0.0093355	-0.0059541	-0.0110182	-6.12E-06
A_23_P26895	TUBD1	**-0.0058598**	-0.0046933	-0.0020756	-0.0040502	-1.39E-06
A_23_P13797	C12orf49	**-0.00692**	-0.0046471	-0.0017332	-0.0067706	-3.36E-06

A_23_P109420	BMS1	**-0.0066379**	**-0.006063**	-0.0026631	-0.0061718	-4.65E-06
A_24_P101629	FAM127B	**-0.0077941**	**-0.0075366**	-0.0021754	-0.0060775	-3.40E-06

SI Table 4: List of 78 metabolic features significantly associated with at least one TRAP exposure, their beta coefficients, cluster memberships, and annotations.

List of all 78 metabolic features that were found as significantly associated to at least one TRAP in our MVN model, after Bonferroni correction of the number of metabolic features (N=6,040), with their corresponding Beta coefficients and Annotations, and the percentage of missing values before imputation in the dataset (N=295 observations). Bold beta coefficients indicate the significant features. In case more than one feature was associated with the same metabolite (different adducts, in-source fragments), an asterisk after the metabolite name indicates the feature with highest intensity.

			Regression coefficients						
Metabolite ID	Percentage missing	Number of associated TRAP	PM2.5	PM10	NO2	BC	PCNT	Cluster member-ship	Metabolite name
194.9746@2.4935539	27.46	1	0.006	-0.001	**0.031**	0.012	1.00E-05	1	
191.0982@2.8594065	21.36	1	-0.01	0.001	**-0.022**	0.015	-6.60E-07	2	
1080.6346@6.914152	16.61	1	-0.01	-0.009	**-0.018**	0.014	2.20E-06	3	
278.0331@3.1873538	14.24	1	0.003	0.002	**0.019**	-0.02	-4.90E-06	4	
375.0063@3.190292	14.24	1	-0.001	-0.002	**0.016**	-0.014	-5.30E-06	5	
310.1447@7.194015	13.9	1	-0.012	-0.007	**0.032**	-0.023	-9.20E-06	6	
800.9799@6.9184003	11.53	1	-0.001	0	**-0.025**	0.001	-6.90E-06	7	
526.3199@6.5894794	11.19	1	0.004	0.002	**0.018**	-0.022	-1.10E-05	8	
275.1731@2.4011953	11.53	1	-0.004	-0.001	**-0.011**	0.009	6.90E-07	9	
364.1552@7.163959	8.47	1	0.003	0	**0.017**	-0.013	-5.70E-06	10	
621.9284@6.8350163	3.39	1	0.001	-0.002	**0.011**	-0.01	-7.10E-06	11	
211.0606@4.565889	3.73	1	0.007	0.003	**0.012**	-0.006	-3.00E-06	12	Indole-3-propionic acid 1
189.0787@4.5659895	0.68	1	0.004	0.001	**0.008**	-0.007	-3.20E-06	12	Indole-3-propionic acid *
194.0802@3.189687	0	1	0	-0.001	**0.016**	-0.013	-1.90E-06	13	Caffeine *
216.0619@3.190204	0.68	1	0.002	0	**0.016**	-0.013	-2.50E-06	13	Caffeine 1
325.0236@3.1906562	1.02	1	0.002	0	**0.018**	-0.013	-1.40E-06	13	
329.2565@5.3163505	1.69	1	-0.003	-0.002	**0.011**	-0.022	-8.70E-06	14	Undecanoylcarnitine (C11:0)
249.0319@2.0463903	0.34	1	0	-0.001	**-0.005**	0.007	3.90E-06	15	
329.2392@5.940575	0.34	1	-0.002	0.001	**-0.008**	0.017	5.40E-06	16	
817.9539@6.9155455	0.68	1	0.006	0.005	**-0.013**	0.025	6.30E-06	17	
180.0644@2.6812208	0.34	1	0.003	0	**0.012**	-0.014	-5.30E-06	18	Paraxanthine
311.0078@2.6815634	0.68	1	0.003	0	**0.011**	-0.005	-5.80E-07	18	
275.1732@2.2214637	0.34	1	-0.003	-0.002	**-0.009**	0.005	3.10E-06	19	
284.9736@1.7443023	0.68	1	0.002	0.002	**-0.009**	0.006	3.80E-07	20	
427.3655@6.4506593	0	1	-0.001	0	**-0.006**	0.005	1.20E-07	21	Stearoylcarnitine (C18:0)
411.3015@6.6322703	0	1	0.001	0	**0.005**	-0.003	6.10E-08	22	
645.9504@6.740771	0	1	0.002	0	**0.006**	-0.005	-1.40E-06	23	
318.1474@6.836559	1.36	1	0.002	0	**0.007**	-0.011	-4.20E-06	24	
550.3041@6.912425	0	1	0.001	0.001	**-0.01**	0.015	4.00E-06	25	
227.0561@3.8431869	0	1	0.002	0.001	**0.009**	-0.009	-3.50E-06	26	Indolelactic acid 1
205.0735@3.8432918	0	1	0.002	0.001	**0.011**	-0.013	-2.70E-06	26	Indolelactic acid *

301.2077@5.6644535	0	1	-0.003	-0.001	**-0.008**	0.005	1.90E-06	27	
301.2068@5.671208	0	1	-0.003	-0.001	**-0.009**	0.003	4.10E-07	27	
1038.6552@6.9790444	0	1	-0.001	0.001	**-0.016**	0.03	6.40E-06	28	
382.1966@5.165206	1.02	2	-0.001	-0.006	**0.014**	**-0.042**	-1.80E-05	29	
296.0202@2.0461934	0	1	0.001	0.002	**-0.007**	0.005	2.60E-06	30	
134.0727@2.0469444	0	1	0.005	0.004	**-0.008**	0.012	5.70E-06	31	
726.0012@6.8730736	0.34	1	0.001	-0.001	**0.006**	-0.008	-4.20E-06	32	
773.9645@7.0033507	0.34	1	0.001	0	**-0.006**	0.008	2.60E-06	33	
119.0738@2.0438619	0	1	0.003	0.003	**-0.006**	0.008	3.10E-06	34	
102.0469@2.045386	0	1	0.002	0.002	**-0.006**	0.008	3.90E-06	34	
166.0825@2.0461037	0	1	0.001	0.002	**-0.006**	0.008	3.80E-06	34	
102.0467@2.046496	0	1	0.002	0.002	**-0.005**	0.008	3.70E-06	34	Phenylalanine 1
360.1938@5.0027375	0.68	1	0	-0.001	**0.013**	-0.012	-4.10E-06	35	Cortisone
549.9001@6.96992	0	1	0.004	0.002	**0.004**	0	1.50E-06	36	
279.1467@2.756788	0	1	-0.004	0.001	**-0.013**	0.015	6.80E-06	37	
389.2648@7.4307146	0.34	1	0	0	**-0.005**	0.003	8.10E-07	38	
206.0381@1.7446203	0.34	1	0	0.003	**-0.011**	0.018	5.40E-06	39	
285.1935@3.928641	0	1	0	0.002	**-0.009**	0.015	5.20E-06	40	Octenoylcarnitine (C8:1)
309.1936@4.3509645	0	1	0	0.002	**-0.008**	0.013	6.00E-06	41	Octanoylcarnitine (C8:0)
315.2412@5.1233363	0	1	-0.004	-0.004	**0.021**	-0.033	-1.40E-05	42	Decanoylcarnitine (C10:0)
362.2091@5.163903	0	1	-0.002	-0.006	**0.017**	-0.028	-5.60E-06	43	Cortisol *
384.1925@5.165229	0	1	-0.001	-0.006	**0.014**	-0.027	-8.60E-06	43	Cortisol 1
509.3399@6.7000976	0	1	0.001	0	**0.006**	-0.005	-9.40E-07	44	
484.3444@6.7130146	0	1	0.001	0	**0.004**	-0.004	-2.00E-06	45	
547.3482@6.8921647	0.68	1	0.001	0	**0.01**	-0.013	-5.20E-06	46	
85.0893@1.3623636	0	1	0.003	0.004	**-0.007**	0.009	3.70E-06	47	Isoleucine 1
131.0946@1.3639117	0	1	0.004	0.005	**-0.007**	0.011	4.90E-06	47	Isoleucine *
471.647@6.806502	0	1	0.002	0.001	**0.005**	-0.009	-3.40E-06	48	
119.0736@2.0465102	0	1	-0.001	0	**-0.006**	0.007	2.80E-06	49	Phenylalanine 2
318.1503@7.112757	0	1	0.002	0.001	**0.007**	0	4.70E-07	50	
482.2165@7.197076	0	1	-0.002	-0.001	**-0.004**	-0.001	-2.10E-07	51	
482.2173@7.197173	0	1	-0.001	0	**-0.005**	0	-5.80E-08	51	
587.2991@6.897131	0	1	0	0	**-0.005**	0.007	1.20E-06	52	
165.0795@2.0396664	0	1	0.001	0.001	**-0.006**	0.007	2.90E-06	53	
165.0795@2.0465238	0	1	0.001	0.002	**-0.006**	0.006	2.70E-06	53	Phenylalanine *
477.2857@6.9057713	0	1	0	-0.001	**-0.009**	0.017	1.60E-06	54	LysoPE(18:2)
390.2372@7.195364	0	1	0	0	**0.002**	-0.001	-1.90E-07	55	
644.5358@7.188575	10.85	2	-0.005	-0.006	-0.001	**-0.039**	**-1.70E-05**	56	
606.5602@8.765215	10.17	1	-0.005	-0.008	0.004	**-0.032**	-9.00E-06	56	
331.2644@8.786948	10.17	1	-0.006	-0.007	0.002	**-0.038**	-1.30E-05	56	
356.1455@7.128134	0	1	-0.004	-0.005	0.009	**-0.029**	-9.80E-06	57	
277.074@3.991694	0.68	1	0.001	0.001	0.001	0.011	**9.40E-06**	58	
1190.2365@6.6071267	1.36	1	0.002	0.003	0	0.022	**1.20E-05**	59	

418.1757@6.6988316	0	1	0.01	0.01	-0.004	0.028	**1.90E-05**	60
1015.6548@7.0011153	8.47	2	**-0.024**	**-0.021**	-0.005	-0.006	-4.90E-06	61
429.7421@8.575909	7.12	2	**-0.027**	**-0.027**	-0.002	0.003	2.60E-06	62
156.0763@2.4506986	4.07	1	0.017	**0.019**	0.001	0.016	4.70E-07	63

SI Table 5. Details for annotation of metabolic features, including ion species, mass-to-charge ratio (*m/z*) and retention time for each feature, the percentage of missing values before imputation in the dataset (N=295 observations), and the level of annotation confidence according to the MSI scale. In case more than one feature was associated with the same metabolite (different adducts, in-source fragments), an asterisk after the metabolite name indicates the feature with highest intensity.

Metabolite ID	Percentage missing	Retention time (min)	*m/z*	MSI level of confidence	Ion	Metabolite name	Cluster
85.0893@1.3623636	0	1.3624	86.09658	1	$[M-CH2O2+H]^+$	Isoleucine 1	47
131.0946@1.3639117	0	1.3639	132.1019	1	$[M+H]^+$	Isoleucine *	47
284.9736@1.7443023	0.68	1.7443	285.9809				20
206.0381@1.7446203	0.34	1.7446	207.0454				39
165.0795@2.0396664	0	2.0397	166.0868				53
119.0738@2.0438619	0	2.0439	120.0811				34
102.0469@2.045386	0	2.0454	103.0542				34
166.0825@2.0461037	0	2.0461	167.0898				34
296.0202@2.0461934	0	2.0462	297.0275				30
249.0319@2.0463903	0.34	2.0464	250.0392				15
102.0467@2.046496	0	2.0465	103.054	1	$[M-CH5NO2+H]^+$	Phenylalanine 1	34
119.0736@2.0465102	0	2.0465	120.0809	1	$[M-CH2O2+H]^+$	Phenylalanine 2	49
165.0795@2.0465238	0	2.0465	166.0868	1	$[M+H]^+$	Phenylalanine *	53
134.0727@2.0469444	0	2.0469	135.08				31
275.1732@2.2214637	0.34	2.2215	276.1805				19
275.1731@2.4011953	11.53	2.4012	276.1804				9
156.0763@2.4506986	4.07	2.4507	157.0836				63
194.9746@2.4935539	27.46	2.4936	195.9819				1
180.0644@2.6812208	0.34	2.6812	181.0717	1	$[M+H]^+$	Paraxanthine	18
311.0078@2.6815634	0.68	2.6816	312.0151				18
279.1467@2.756788	0	2.7568	280.154				37
191.0982@2.8594065	21.36	2.8594	192.1055				2
278.0331@3.1873538	14.24	3.1874	279.0404				4
194.0802@3.189687	0	3.1897	195.0875	1	$[M+H]^+$	Caffeine *	13
216.0619@3.190204	0.68	3.1902	217.0692	1	$[M+Na]^+$	Caffeine 1	13
375.0063@3.190292	14.24	3.1903	376.0136				5
325.0236@3.1906562	1.02	3.1907	326.0309				13
227.0561@3.8431869	0	3.8432	228.0634	1	$[M+Na]^+$	Indolelactic acid 1	26
205.0735@3.8432918	0	3.8433	206.0808	1	$[M+H]^+$	Indolelactic acid *	26
285.1935@3.928641	0	3.9286	286.2008	2	$[M+H]^+$	Octenoylcarnitine (C8:1)	40
277.074@3.991694	0.68	3.9917	278.0813				58
309.1936@4.3509645	0	4.351	310.2009	2	$[M+Na]^+$	Octanoylcarnitine (C8:0)	41
211.0606@4.565889	3.73	4.5659	212.0679	1	$[M+Na]^+$	Indole-3-propionic acid 1	12
189.0787@4.5659895	0.68	4.566	190.086	1	$[M+H]^+$	Indole-3-propionic acid *	12

360.1938@5.0027375	0.68	5.0027	361.2011	1	$[M+H]^+$	Cortisone	35
315.2412@5.1233363	0	5.1233	316.2485	1	$[M+H]^+$	Decanoylcarnitine (C10:0)	42
362.2091@5.163903	0	5.1639	363.2164	1	$[M+H]^+$	Cortisol *	43
382.1966@5.165206	1.02	5.1652	383.2039				29
384.1925@5.165229	0	5.1652	385.1998	1	$[M+Na]^+$	Cortisol 1	43
329.2565@5.3163505	1.69	5.3164	330.2638	2	$[M+H]^+$	Undecanoylcarnitine (C11:0)	14
301.2077@5.6644535	0	5.6645	302.215				27
301.2068@5.671208	0	5.6712	302.2141				27
329.2392@5.940575	0.34	5.9406	330.2465				16
427.3655@6.4506593	0	6.4507	428.3728	2	$[M+H]^+$	Stearoylcarnitine (C18:0)	21
526.3199@6.5894794	11.19	6.5895	527.3272				8
1190.2365@6.6071267	1.36	6.6071	1191.244				59
411.3015@6.6322703	0	6.6323	412.3088				22
645.9504@6.740771	0	6.7408	646.9577				23
484.3444@6.7130146	0	6.713	485.3517				45
418.1757@6.6988316	0	6.6988	419.183				60
509.3399@6.7000976	0	6.7001	510.3472				44
471.647@6.806502	0	6.8065	472.6543				48
549.9001@6.96992	0	6.9699	550.9074				36
726.0012@6.8730736	0.34	6.8731	727.0085				32
621.9284@6.8350163	3.39	6.835	622.9357				11
318.1474@6.836559	1.36	6.8366	319.1547				24
547.3482@6.8921647	0.68	6.8922	548.3555				46
587.2991@6.897131	0	6.8971	588.3064				52
477.2857@6.9057713	0	6.9058	478.293	2	$[M+H]^+$	LysoPE(18:2)	54
550.3041@6.912425	0	6.9124	551.3114				25
1080.6346@6.914152	16.61	6.9142	1081.642				3
800.9799@6.9184003	11.53	6.9184	801.9872				7
817.9539@6.9155455	0.68	6.9155	818.9612				17
1038.6552@6.9790444	0	6.979	1039.662				28
1015.6548@7.0011153	8.47	7.0011	1016.662				61
773.9645@7.0033507	0.34	7.0034	774.9718				33
318.1503@7.112757	0	7.1128	319.1576				50
356.1455@7.128134	0	7.1281	357.1528				57
310.1447@7.194015	13.9	7.194	311.152				6
364.1552@7.163959	8.47	7.164	365.1625				10
644.5358@7.188575	10.85	7.1886	645.5431				56
390.2372@7.195364	0	7.1954	391.2445				55
482.2165@7.197076	0	7.1971	483.2238				51
482.2173@7.197173	0	7.1972	483.2246				51
389.2648@7.4307146	0.34	7.4307	390.2721				38
429.7421@8.575909	7.12	8.5759	430.7494				62
606.5602@8.765215	10.17	8.7652	607.5675				56

331.2644@8.786948	10.17	8.7869	332.2717	56

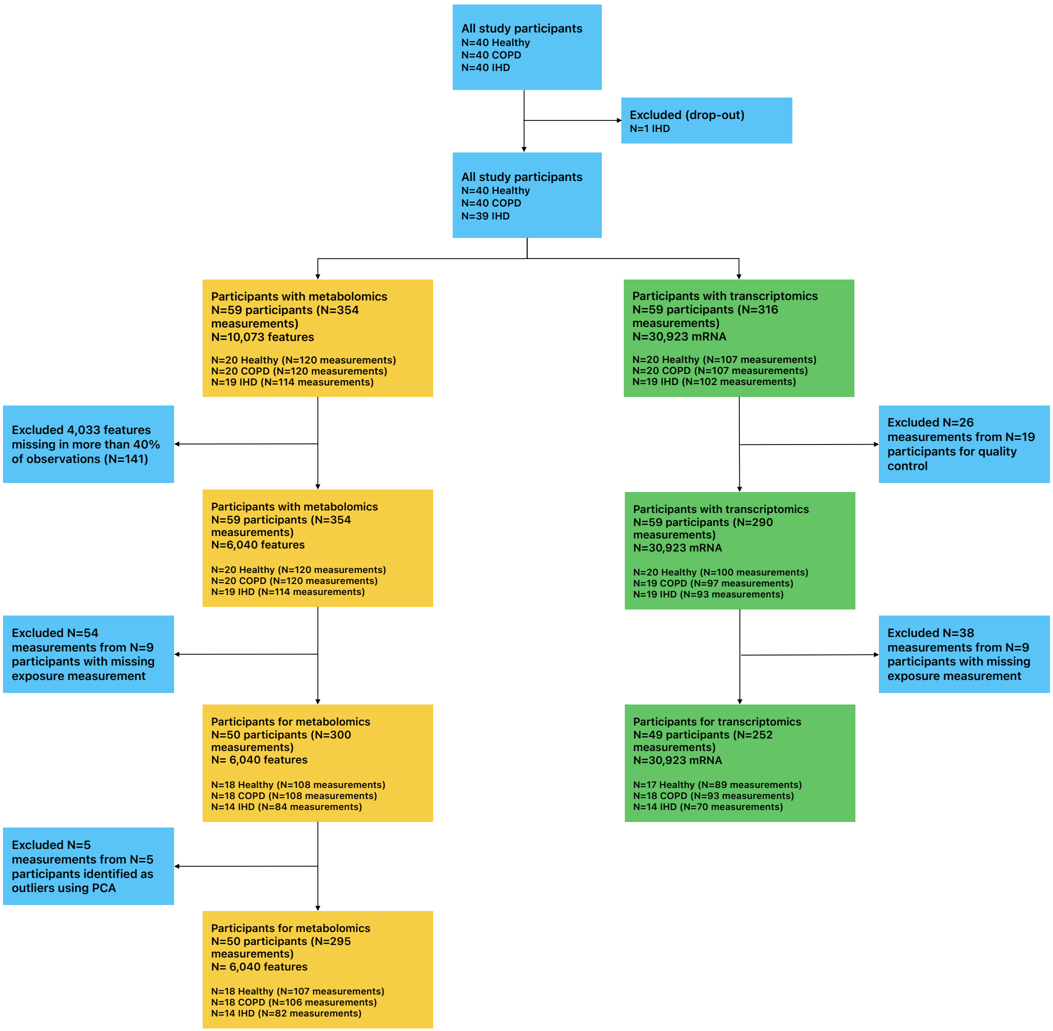

SI Figure S1: Schematic representation of the study population and exclusion criteria.

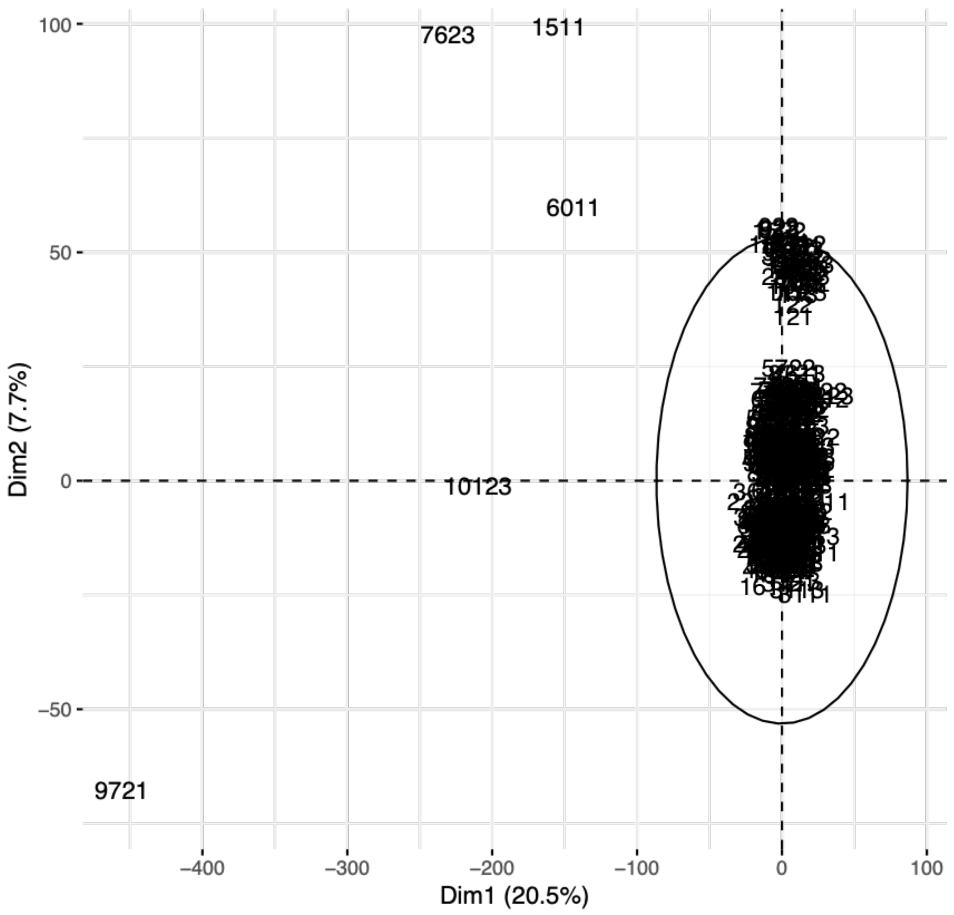

SI Figure S2: Score plots of the first (x-axis) and second (y-axis) principal components from principal component analysis (PCA) applied to the 300 metabolic profiles collected for the 50 participants. The 5 outlying observations: 9721, 7623, 1511, 6011 and 10123 were excluded for subsequent analysis.

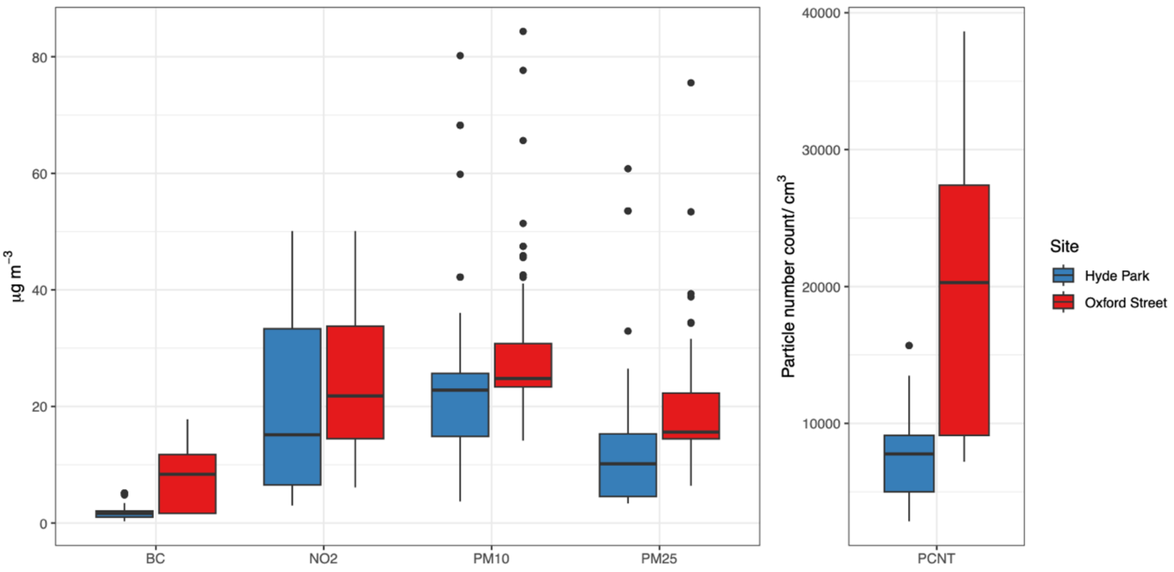

SI Figure S3: Traffic Related Air Pollutant (TRAP) concentrations at both locations in the Oxford Street II study (values measured in Hyde Park are in blue, and red for Oxford Street). Concentrations are expressed in $\mu g/m^3$ for all TRAPs except PCNT which is expressed in particle number count/cm^3.

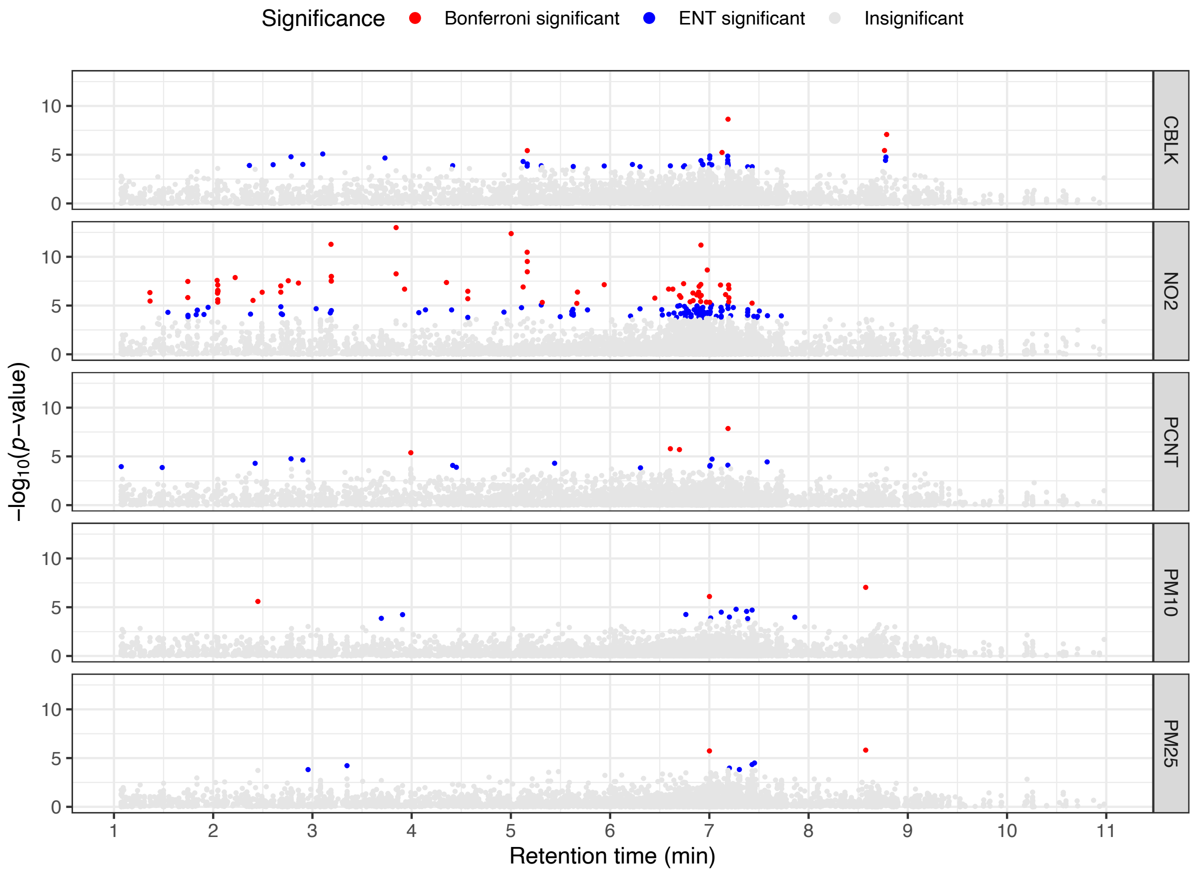

SI Figure S4: Manhattan Plots illustrating the association between each metabolic feature and each individual TRAP using our univariate analysis. Each metabolic feature is represented by its retention time (minutes, X-axis) and its -log10 p-value (Y-axis). Results are presented for each TRAP exposure, separately and metabolic features associated with TRAP exposures at a Bonferroni significance level correcting for ENT=284 and 6,040 tests are represented in blue and red, respectively.

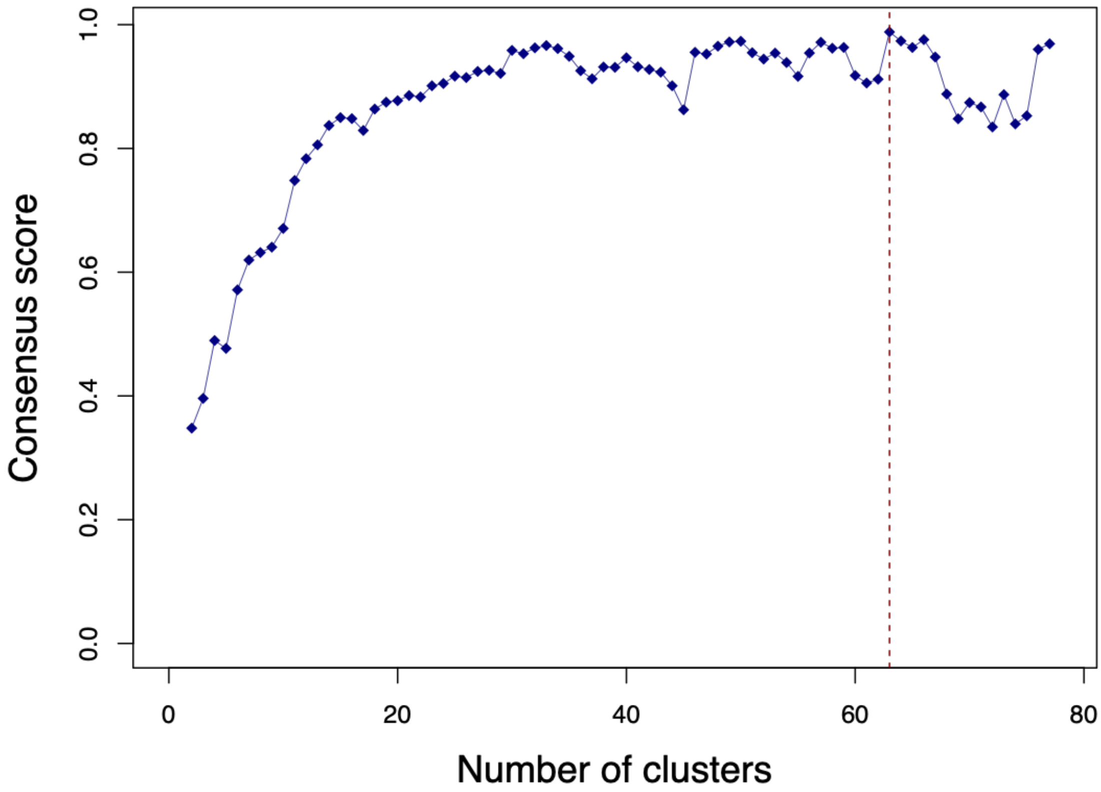

SI Figure S5: Calibration plot for consensus clustering of the 78 metabolic features selected with the MVN model as significantly associated to TRAP exposure. The stability score was plotted against the number of clusters ranging from n= 2 to n= 77. The highest stability score was achieved at 63 clusters (red dotted line).

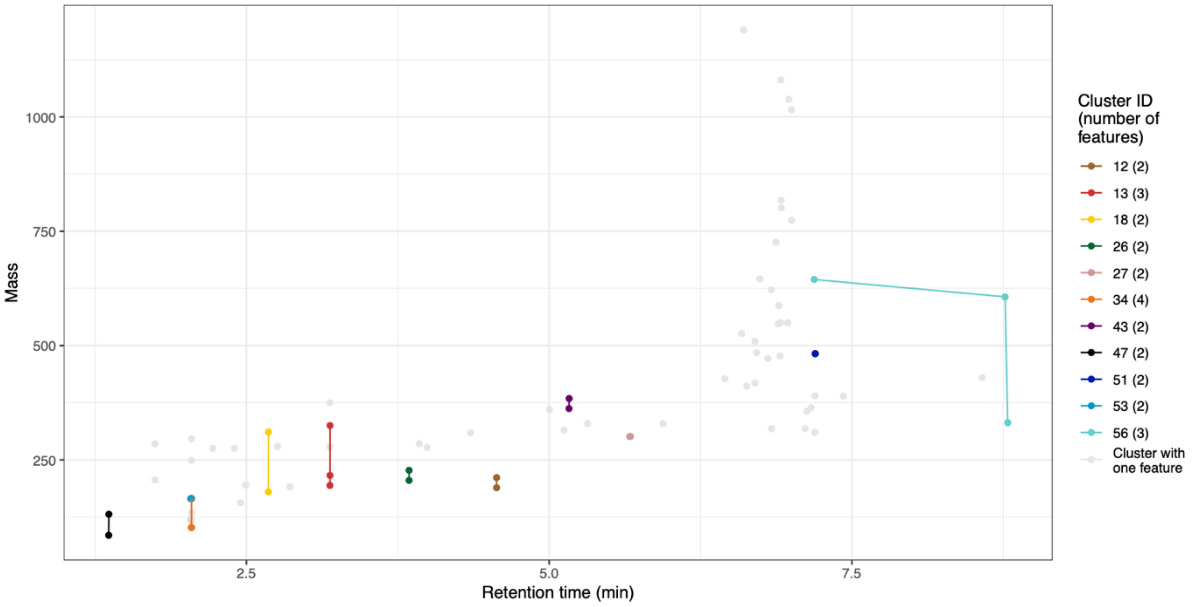

SI Figure S6: Scatter plot of molecular mass (Y axis) as a function of retention time (in minutes, X axis) for the 78 metabolic features found associated with at least on TRAP exposure. Features that have been clustered together are coloured by their cluster ID and united by a line, the colours are detailed in the legend. Clusters of single features are plotted in light grey.

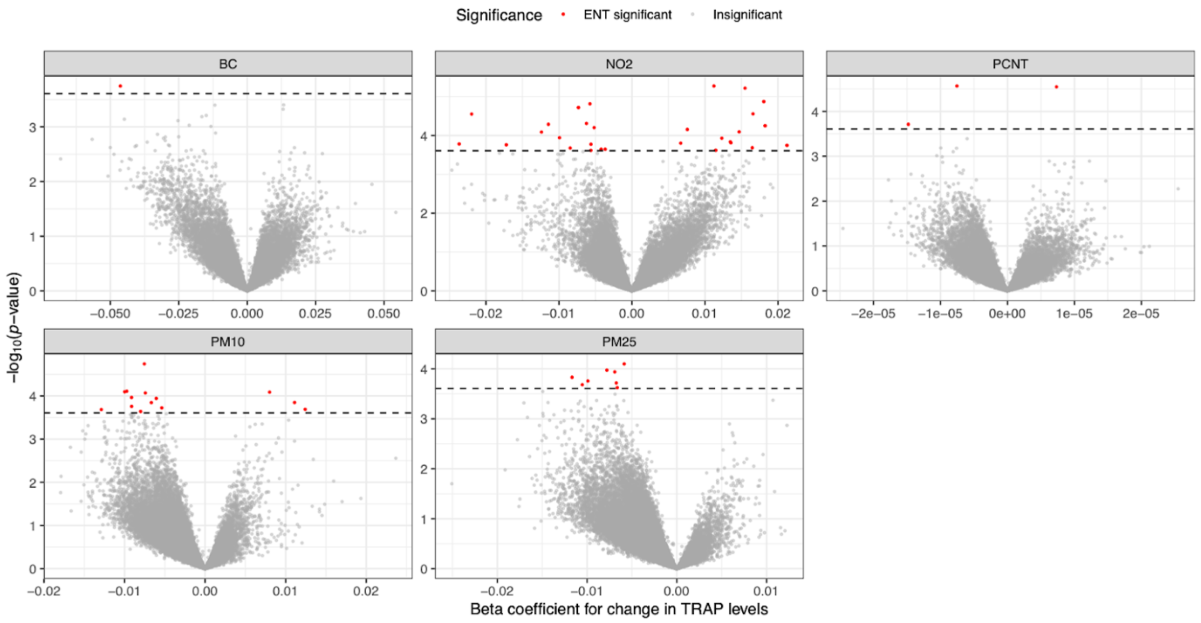

SI Figure S7: Volcano plots illustrating the -$\log_{10}$ *p*-values of each mRNA plotted against their beta coefficients in MVN models for each TRAP exposure. mRNAs coloured in red are significant after adjusting for effective number of tests (ENT, n=202).

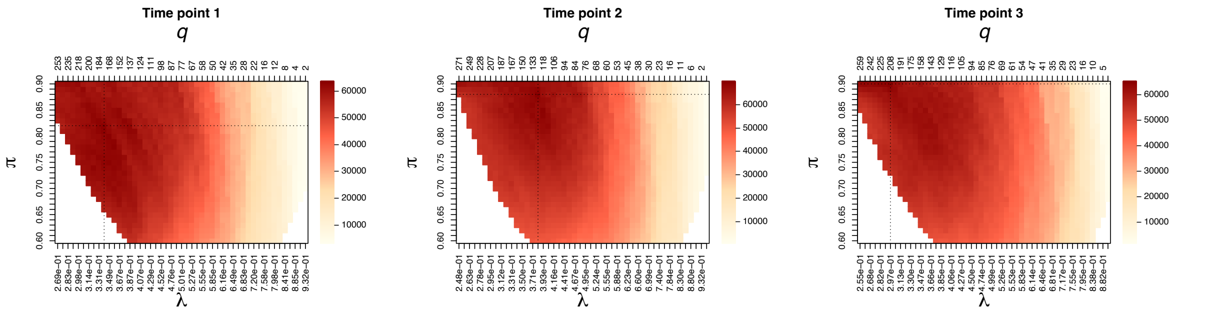

SI Figure S8: Calibration plots for metabolomics networks, for the three time points, 2 h before walks (time point 1), 2 h (time point 2) and 24 h (time point 3) after walks. The colour denotes the stability score for each combination of the two hyperparameters: penalisation (λ) and threshold of selection proportion (π).

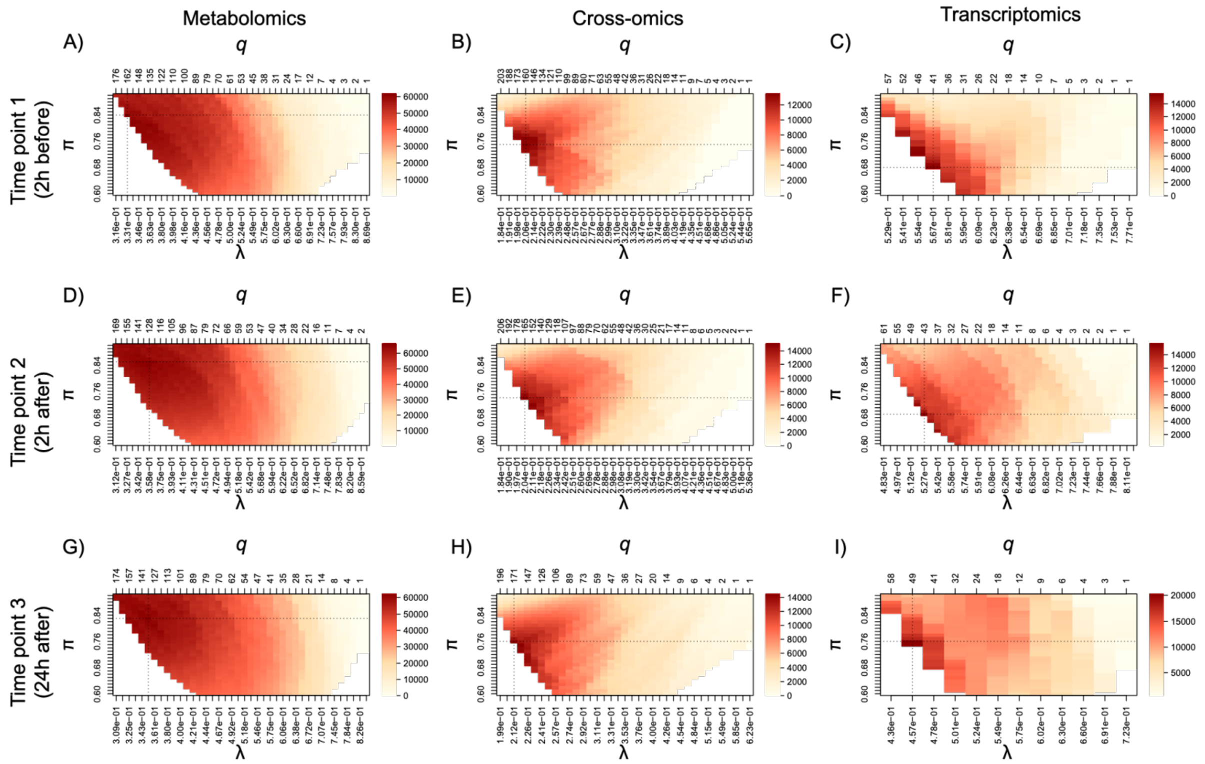

SI Figure S9: Calibration plots for multi-omic networks, for the three time points, 2 h before walks (time point 1), 2 h (time point 2) and 24 h (time point 3) after walks. At each time point, a calibration plot is shown for each block: metabolomics only, between metabolomics and transcriptomics, and transcriptomics only. The colour denotes the stability score for each combination of the two hyperparameters: penalisation (λ) and threshold of selection proportion (π).
